# A comprehensive analysis of induced pluripotent stem cell (iPSC) production and applications

**DOI:** 10.3389/fcell.2025.1593207

**Published:** 2025-05-08

**Authors:** Margarita Matiukhova, Anastasia Ryapolova, Vladimir Andriianov, Vasiliy Reshetnikov, Sophia Zhuravleva, Roman Ivanov, Alexander Karabelsky, Ekaterina Minskaia

**Affiliations:** Translational Medicine Research Center, Sirius University of Science and Technology, Sochi, Russia

**Keywords:** induced pluripotent stem cells, iPSC, transcription factors, cell reprogramming, cell therapy, regenerative medicine, *in vitro* disease modeling, viral delivery

## Abstract

The ability to reprogram mature, differentiated cells into induced pluripotent stem cells (iPSCs) using exogenous pluripotency factors opened up unprecedented opportunities for their application in biomedicine. iPSCs are already successfully used in cell and regenerative therapy, as various drug discovery platforms and for *in vitro* disease modeling. However, even though already 20 years have passed since their discovery, the production of iPSC-based therapies is still associated with a number of hurdles due to low reprogramming efficiency, the complexity of accurate characterization of the resulting colonies, and the concerns associated with the safety of this approach. However, significant progress in many areas of molecular biology facilitated the production, characterization, and thorough assessment of the safety profile of iPSCs. The number of iPSC-based studies has been steadily increasing in recent years, leading to the accumulation of significant knowledge in this area. In this review, we aimed to provide a comprehensive analysis of methods used for reprogramming and subsequent characterization of iPSCs, discussed barriers towards achieving these goals, and various approaches to improve the efficiency of reprogramming of different cell populations. In addition, we focused on the analysis of iPSC application in preclinical and clinical studies. The accumulated breadth of data helps to draw conclusions about the future of this technology in biomedicine.

## 1 History of cell reprogramming

In 1962, John Gurdon laid the foundation for reprogramming by demonstrating that a somatic cell nucleus transferred into an enucleated egg could revert to a pluripotent state (Gurdon, 1962). In 1996, Ian Wilmut and colleagues were the first to clone a mammal, Dolly the sheep, using the same somatic cell nuclear transfer (SCNT) principle (Wilmut et al., 1997). This technology proved that the somatic cell nucleus contains the genetic information needed to revert to a pluripotent state and that the egg contains factors capable of regulating the gene expression profile that mediates the transition to a pluripotent state (Bailly et al., 2022). In 2001, the new reprogramming approach relying on the fusion of somatic cells with embryonic stem cells (ESCs) showed that embryonic stem cells also contain reprogramming factors (Tada et al., 2001). In 2006, Shinya Yamanaka and his team screened twenty-four transcription factors (TFs) and found that overexpression of four of them, Oct4, Sox2, Klf4, and c-Myc (the so-called “Yamanaka factors”), allows reprogramming of mouse fibroblasts into cells similar to embryonic stem cells (Takahashi K. and Yamanaka, 2006). These cells were called induced pluripotent stem cells (iPSCs). In 2007, the experiment was successfully reproduced with human fibroblasts (Takahashi K. et al., 2007). The number of studies using iPSCs or aiming to improve the reprogramming efficiency has been steadily increasing ever since (Kobold et al., 2023). Non-integrative approaches using virus-free delivery systems can be considered the most promising methods for increasing the biosafety of iPSCs. The steady development of molecular biology methods increased the accuracy of the characterization of the obtained iPSCs, including the assessment of possible genomic changes, and facilitated the detailed analysis of the differentiated cells. This work has not remained in vain, and already 10 years ago, the first clinical trials of iPSC-based therapies for the treatment of age-related macular degeneration were started (Mandai et al., 2017). Several dozen iPSC-based cell products are currently in various phases of clinical trials (Hui and Yamanaka, 2024; Kobold et al., 2023). iPSCs can be used to treat various groups of diseases, including retinopathies, cardiovascular and neurodegenerative diseases, as well as oncological diseases. Existing clinical trials mainly use ready-made HLA-matched allogeneic (donor) instead of autologous iPSCs, which can significantly reduce both the time and costs of therapy production (Normile, 2018). Such donor iPSCs are stored in specialized cell banks, the number of which is growing together with the demand for iPSC-based products (Mah et al., 2023). For example, the Kyoto University iPSC Research and Application Center, led by Yamanaka, is developing an iPSC bank where 75 lines could cover 80% of the Japanese population through HLA matching. Donor iPSCs may be safer than cells from elderly patients and allow for rapid production of ready-to-use cell products (Liu A. et al., 2020; Tsai et al., 2011; Normile, 2018). However, there will still be a demand for patient-specific (autologous) iPSCs, especially for the purpose of screening adverse drug reactions (Normile, 2018).

The aim of this review was to discuss recent advances in the field of iPSC production and characterization, and their preclinical and clinical applications. In addition, we touched upon the prospects and limitations of using donor iPSC biobanks.

## 2 Factors and mechanisms of pluripotency

Reprogramming of mature differentiated cells to a state of pluripotency is achieved through ectopic expression of specific transcription factors, ensuring the transition of the cell to a pluripotent state. At the early stages of reprogramming, the coordinated action of these exogenous factors suppresses the expression of genes specific to somatic cells, and at later stages, activates the endogenous expression of pluripotency factors. These changes in gene expression ensure the reprogramming of somatic cells, which acquire features characteristic of ESCs (Han et al., 2021; Karami et al., 2023).

The expression of exogenous reprogramming factors does not necessarily have to be maintained continuously, since they gradually activate a self-reinforcing “pluripotency network” via the expression of endogenous factors, which maintains the global pattern of embryonic gene expression (Bayart and Cohen-Haguenauer, 2013). It is believed that the initiation of early reprogramming events is the most complex step associated with the inefficient access of exogenous TFs to closed chromatin, while late events are likely to be more deterministic and hierarchical (Cerneckis et al., 2024). The process of cell reprogramming is associated with remodeling of chromatin structure and changes in the epigenome, as well as changes in almost all aspects of cell biology, including metabolism, cell signaling, intracellular transport, proteostasis, and others (Borkent et al., 2016; Buckley et al., 2012; Cerneckis et al., 2024; Qin et al., 2014; Simic et al., 2019; Wu et al., 2019).

Traditional combinations of transcription factors for somatic cell reprogramming, the “Yamanaka cocktail,” consist of four factors: Oct4, Sox2, Klf4, and c-Myc (OSKM). At the initial stage of reprogramming, c-Myc associates with histone acetyltransferase complexes and induces global histone acetylation, which ensures the binding of exogenous Oct4 and Sox2 to their specific target loci (Soufi et al., 2012). c-Myc promotes enhanced reprogramming because the number of its binding sites far exceeds the number of such sites for Oct4 and Sox2 (Takahashi and Yamanaka, 2006). Oct4 and Sox2 are considered to be key TFs that inhibit the expression of genes associated with ESC differentiation (Gillis et al., 2011). Importantly, the expression levels of Sox2 and Oct4 during the reprogramming of somatic cells into iPSCs are critical, as reported in several studies (Gillis et al., 2011; Matsuoka et al., 2012; Yamaguchi et al., 2011), and their specific ratio affects the reprogramming efficiency and the quality of iPSC colonies (Fus-Kujawa et al., 2021). The Klf4 factor plays a dual role throughout the process: on one hand promoting the suppression of the expression of a large number of genes specific to intermediate reprogrammed cells, and on the other hand, inducing the activation of the expression of genes associated with pluripotency (Kulcenty et al., 2015).

Various studies aimed to optimize the “Yamanaka cocktail” for more efficient production of iPSCs with the desired characteristics. In particular, researchers question the need to use the proto-oncogene c-Myc. During the first few days of initial reprogramming, c-Myc enhances the process (Sridharan et al., 2009; Stadtfeld and Hochedlinger, 2010), however, it induces cell proliferation and causes a transition to energy metabolism typical of cancer cells at later stages (Mikkelsen et al., 2008; Sridharan et al., 2009; Stadtfeld and Hochedlinger, 2010). The OSKM combination is not the only option for cellular reprogramming: in the same year as the Yamanaka group, another group led by James Thomson discovered another combination of four genes, Oct4, Sox2, Nanog, and Lin28 (OSNL), sufficient to reprogram human somatic cells into pluripotent stem cells. Nanog functions as one of the essential factors for maintaining pluripotency along with Oct4 and Sox2 (Boyer et al., 2005; Loh et al., 2006), but is not critical for the generation of iPSC clones in general (Takahashi and Yamanaka, 2006). Lin28 is likely to exert similar effects as c-Myc, since it also affects the early phase of iPSC generation by accelerating cell proliferation (Golipour et al., 2012). Together, Nanog and Lin28 are effective analogs of Klf4 and c-Myc (Liu G. et al., 2020). The absence of Lin28 or Nanog in the reprogramming factor cocktail was shown to cause a decrease in the number of iPSC colonies (Fus-Kujawa et al., 2021; Gillis et al., 2011; Wang et al., 2019; Yu et al., 2007).

Attempts were also made to use traditional combinations of TFs at different ratios or to vary the combination of factors in order to increase the efficiency of reprogramming (Bailly et al., 2022; Lapasset et al., 2011; Liao et al., 2008). For example, a combination of six OSKMNL factors did not only promote a 10-fold increase in the efficiency of fibroblast reprogramming compared to the OSNL combination but also allowed for the successful reprogramming of fibroblasts obtained from old donors (Bailly et al., 2022; Lapasset et al., 2011; Liao et al., 2008). On the other hand, in addition to attempts to increase the efficiency of reprogramming by increasing the number of delivered factors, some studies are aimed, on the contrary, at minimizing their number and, therefore, reducing the load on the reprogrammed cell. For example, Feng and colleagues (Feng et al., 2009b) developed a three-factor combination containing the transcription factor Esrrb, which, together with Oct4 and Sox2, allows reprogramming of mouse embryonic fibroblasts (MEFs) into iPSCs with greater efficiency than the “Yamanaka cocktail”. Subsequently, other combinations were successfully used, including various combinations of two of these (Oct4, Sox2, Klf4, and c-Myc) factors (Huangfu et al., 2008b; Kim et al., 2008). In a number of studies, it was possible to obtain iPSCs using only Oct4 (Kim J. B. et al., 2009a; 2009b; Liu A. et al., 2020; Tsai et al., 2011). These results can probably be applied to cells that already have endogenous expression of other key TFs for reprogramming. For example, melanocytes express Sox2, and for their successful reprogramming into iPSCs, the introduction of exogenous Sox2 is not necessary (Utikal et al., 2009). Similar situations are observed with other cell types; for example, umbilical cord blood stem cells can be reprogrammed only by Oct4 and Sox2 (Giorgetti et al., 2009; Meng et al., 2012), while human neural stem cells only need exogenous Oct4 (Kim J. B. et al., 2009a). Some studies, for example, on mouse fibroblasts, demonstrated that reprogramming can be accomplished even without exogenous Oct4 expression (Bailly et al., 2022; Velychko et al., 2019).

In addition, a number of studies demonstrated that the addition of small molecule inducers of pluripotency can also improve reprogramming efficiency, compensate for or enhance the action of individual TFs (Rehman et al., 2024). For example, reprogramming efficiency is increased by the addition of certain groups of compounds: DNA methyltransferase inhibitors such as 5-azacytidine (Aza) (Mikkelsen et al., 2008), histone deacetylase inhibitors such as valproic acid (VPA) (Huangfu et al., 2008a) or sodium butyrate (Mali et al., 2010), or histone demethylase inhibitors such as parnate (Li H. et al., 2009).

## 3 Reprogramming methods

**FIGURE 1:**
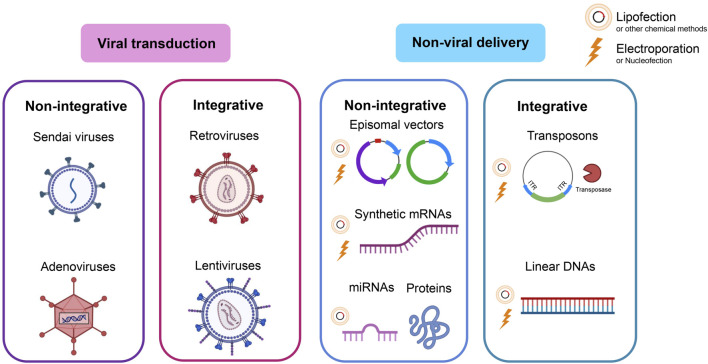
Reprogramming methods. Classical methods deliver exogenous TFs via viral transduction or non-viral delivery. In both cases, either the option of integrating the DNA coding for pluripotency factors into the host cell genome (integrative methods) or a transient option that does not rely on integration (non-integrative methods) is possible.

This section describes the main reprogramming methods with their advantages and disadvantages. Classical reprogramming methods are aimed at inducing pluripotency by delivering exogenous TFs into the cells via viral transduction or non-viral delivery. In both cases, the two options are possible: stable expression as a result of TF-encoding DNA integration into the host cell genome (integrative methods) or transient expression (non-integrative methods) (Figure 1).

### 3.1 Viral reprogramming methods

The most common method of reprogramming somatic cells into iPSCs relies on the delivery of exogenous pluripotency factors via viral transduction. The main advantage of virus-based reprogramming is the high efficiency of gene delivery to various types of cells as compared to, for example, transfection, and higher cell viability as compared to electroporation (Cerneckis et al., 2024; Haridhasapavalan et al., 2019; MacArthur et al., 2012; Scesa et al., 2021; Seki et al., 2010; Zhou W. and Freed, 2009). Various methods for obtaining iPSCs using popular viral vectors have already been described in the literature: the integrative retro-(RV)/lentiviruses (LV), and the non-integrative adenoviruses (Ad), adeno-associated viruses (AAV), herpes simplex viruses (HSV), and Sendai viruses (SeV) (Anguela and High, 2019; Giacca and Zacchigna, 2012; Lukashev and Zamyatnin, 2016; Paolini Sguazzi et al., 2021; Pena et al., 2020).

#### 3.1.1 Integrative viruses

##### 3.1.1.1 Retroviral and lentiviral vectors

The first successful method for generating iPSCs from somatic cells relied on the delivery of TFs via non-replicating RVs lacking genome regions coding for proteins required for additional rounds of replication and packaging of the virus (Bayart and Cohen-Haguenauer, 2013). Such RVs can deliver up to 6–8 kb of transgenes and possess high transduction efficiency; however, they are unable to transduce non-dividing cells such as neurons. Later, LVs (a subclass of RVs) began to be used as an alternative safer option. Both are integrative, single-stranded RNA viruses; the transfer of a transgene is carried out via the reverse transcription of their RNA genome into double-stranded DNA, which is then stably integrated into the host cell genome with the help of viral integrase (Paolini Sguazzi et al., 2021). The main difference between LV and RV is that LV has the ability to transduce both dividing and non-dividing cells. The ability to integrate and stably maintain gene expression at a high level during cell divisions plays a significant role in the successful reprogramming, allowing the generation of iPSCs from most cell types (Supplementary Table S1). In addition, LVs can deliver larger transgene sequences than RVs and exhibit higher transduction efficiency in mammalian cells (Bayart and Cohen-Haguenauer, 2013).

The likelihood of insertional mutagenesis and the high and stable expression (which is not necessary) of the delivered TFs, including possible pro-oncogenes c-Myc and Klf4, are the unfortunate disadvantages of using RV and LV vectors (Warren and Lin C., 2019). One way to overcome insertional mutagenesis is to develop vectors that can be removed (inactivated) after integration into the genome using a heterologous recombination system. One such system contains loxP sites in the 3′and 5′LTR regions: after integration into the genome, the expression of Cre recombinase is activated in cells, and non-homologous recombination processes are triggered at loxP sites. The use of this approach led to the generation of human iPSCs free of transgene sequences that are able to maintain their pluripotent state and display a gene expression profile similar to human ESCs (Soldner et al., 2009). Subsequently, a polycistronic LV vector was developed encoding specific reprogramming factors separated by the self-cleaving 2A peptides, resulting in the integration of a single reprogramming cassette with two loxP sites (Chang et al., 2009; Ramos-Mejía et al., 2012). After Cre recombinase-mediated excision, the resulting iPSC lines contain only three LV-derived sequences (loxP site and the regulatory elements). In addition, the use of polycistronic vectors significantly reduces the number of vector copies per cell, which reduces the risk of insertional mutagenesis (Bayart and Cohen-Haguenauer, 2013). Another widely used heterologous recombination system is the Flp/FRT system from *Saccharomyces cerevisiae* (O’Gorman et al., 1991). Despite the lower efficiency compared to the Cre/loxP system (Nakano et al., 2001), it has lower toxicity, which is important when working with primary cells (Schmidt-Supprian and Rajewsky, 2007), and ensures the removal of the transgene after integration (Voelkel et al., 2010). Thus, the use of LV systems with heterologous recombination systems is the most attractive tool for obtaining iPSCs. However, these iPSC cells are still not “genetically pure” pluripotent stem cells (Bayart and Cohen-Haguenauer, 2013).

Another disadvantage of RV systems is the presence of viral promoters in the transgene cassette necessary for efficient expression of TFs during several cell division cycles until pluripotency is achieved. While important at the initial stages, there is no need to maintain the expression of exogenous pluripotency factors indefinitely, since a group of endogenous factors is activated during the reprogramming process. Therefore, expression of these factors in iPSCs is suppressed by the methylation of their promoter regions. However, in the case of LV vectors, complete suppression of expression of integrated transgenes does not always occur leading to the constant expression of TFs (Warren and Lin C., 2019), which can prevent complete cell reprogramming (Hotta and Ellis, 2008). Constitutive activation of reprogramming factors negatively affects the formation of iPSCs and maintains the cells in a state of equilibrium close to pluripotency (Buecker et al., 2010). One of the ways to prevent re-expression of exogenous factors is to control expression using the Tetracycline/doxycycline-inducible system, which ensures transgene repression in the iPSC-like colony and further selection of the completely reprogrammed cells (Bayart and Cohen-Haguenauer, 2013). The success of this approach was demonstrated by qPCR, which confirmed the inactivation of transgenes used for reprogramming and the reactivation of endogenous regulators of pluripotency in the obtained iPSCs.

#### 3.1.2 Non-integrative viruses

##### 3.1.2.1 Adenoviral vectors

One of the methods of non-integrative delivery of exogenous TFs into cells is the use of adenoviral vectors, which lack the ability to replicate (Zhou and Freed, 2009). Ads are DNA viruses that can efficiently deliver genes to both dividing and non-dividing cells *in vitro* and maintain high levels of transgene expression for several days, which allows for successful reprogramming of cells without Ad integration into the host genome. Ads have a broad tissue tropism, which makes them potentially suitable for obtaining iPSCs from various cell types.

The first study describing the successful reprogramming of mouse hepatocytes into iPSCs using the Ad system was published in 2008 by Stadtfeld and colleagues (Stadtfeld et al., 2008b). However, the reprogramming efficiency using Ad vectors was significantly lower than that using LV vectors (Okita et al., 2008; Stadtfeld et al., 2008b). As a possible solution to the problem of low efficiency, it was proposed to create an Ad vector delivering a polycistronic cassette for the expression of all four reprogramming factors (Zhou and Freed, 2009). However, not all Ads are able to deliver a large polycistronic cassette. This hurdle can be overcome by using “gutless” Ads (GLAd), which require a helper Ad, the presence of which greatly complicates the subsequent purification steps (Jozkowicz et al., 2002). To avoid such undesirable consequences, it is possible to use GLAds that do not require a helper virus (“Helper Free” HF-GLAd). However, the use of HF-GLAd has its drawbacks, since it can induce an immune response due to the presence of a capsid structure similar to that of the wild-type Ads and Ads of earlier generations (Muruve et al., 2004). Due to these limitations, both GLAd and HF-GLAd vectors are not used for reprogramming. Despite the fact that Ad vectors are considered non-integrative, their integration is still possible to a certain degree and exceeds the integration of plasmid DNA (Harui et al., 1999), which is also a disadvantage of this system for reprogramming.

##### 3.1.2.2 Sendai virus-based vectors

The Sendai virus-based vector can be used as an alternative to the previously described LV and Ad vectors. SeV is an enveloped, single-stranded, negative-sense RNA paramyxovirus that replicates in the host cell cytoplasm and is eliminated from cells after ∼10 passages post-infection, which is sufficient and safe for successful reprogramming. SeV has a broad cellular tropism since it uses sialic acid as a cellular receptor, which is common in all cell types. The ease of use and high efficiency of SeV-based reprogramming vectors explain their wide application for reprogramming a wide variety of cell types (Supplementary Table S1).

Although the use of SeV-based vectors seems attractive, there are certain limitations: for example, the viral replicase is extremely sensitive to the nature of transgene sequences (Omole and Fakoya, 2018). In addition, SeV is considered difficult to eliminate from the host cells due to its constitutive replication; despite this, it can be eliminated by the 10th passage (MacArthur et al., 2012). Nishimura and colleagues (Nishimura et al., 2011) reported the use of SeV replication-deficient vectors (SeVdp). This improved SeV version mediates persistent transgene expression, while the first-generation recombinant vectors are capable of high but transient transgene expression (Griesenbach et al., 2005). These SeVdp vectors allow for more efficient generation of mouse iPSCs. With the addition of interfering RNAs to the system, SeV genomes can be completely eliminated. Temperature-sensitive SeVs have also been developed, which allows a sharp reduction in the number of vector copies in the cytoplasm by changing the temperature temperature (Nishishita et al., 2012; Ono et al., 2012), while the formed iPSCs are devoid of exogenous nucleic acids (Bayart and Cohen-Haguenauer, 2013).

Optimization of the SeV-based TF delivery system and the high efficiency and safety of such an reprogramming approach (Bhutani et al., 2016; Kunitomi et al., 2022) led to the creation of commercial reprogramming kits (e.g., CytoTune™-iPS 2.0 Sendai Reprogramming Kit), making this system even more popular worldwide.

### 3.2 Non-viral reprogramming methods

Non-viral reprogramming methods include integrative strategies using transposons, non-integrative episomal and minicircle plasmids, and strategies without transgene delivery at all: protein delivery and reprogramming using mRNA.

#### 3.2.1 Non-viral integrative reprogramming methods

##### 3.2.1.1 Transposon-based system

Mobile elements of the genome, represented by DNA fragments - transposons, can be used as a non-viral integrative vector system for the delivery of pluripotency factors (Tipanee et al., 2017). Transposons can change their position using the transposition mechanism: transient expression of transposase allows the transfer of a transgene surrounded by ITRs. The advantage of transposons is the possibility of using longer and more complex transgene sequences. Certain types of transposons are used more frequently: Tol2, Tc1, “Sleeping Beauty” (SB), “Frog Prince,” and transposons of the Piggybac family (PB) (Cherkashova et al., 2020; Davis et al., 2013). The PB transposon system was used by Kaji and colleagues (Kaji et al., 2009) and Woltjen and colleagues (Woltjen et al., 2009): both groups were able to generate human iPSCs from fibroblasts. In these studies, the authors demonstrated traceless removal of exogenous pluripotency factors and scarless removal of the introduced transposon without changing the integration site sequence: this feature is unique to the PB system (Bayart and Cohen-Haguenauer, 2013). The SB system is considered particularly promising, especially the superactive SB100X transposase, which has a 100-fold higher activity in the HeLa cell line compared to the original SB. The efficiency of SB100X-mediated transgene delivery is similar to viral transduction in obtaining both mouse and human iPSCs, but the transposition process leaves some sequences (scars) unlike the original PB system (Bayart and Cohen-Haguenauer, 2013; Cherkashova et al., 2020).

Both systems (PB- and SB-based) allow the removal of the reprogramming cassette and its site-specific exchange via targeted recombination. These features make the transposon/transposase system one of the best options for delivering TFs for reprogramming a wide range of somatic cells to obtain “genetically pure” iPSCs (Bayart and Cohen-Haguenauer, 2013). However, a limitation of transposon-based reprogramming may be the low efficiency of DNA transfection of some primary cell lines. In addition, it should be emphasized that transposition is not always accurate; for example, there are data on changes detected in 5% of transposition cases (Wang et al., 2008). Moreover, due to the uncontrolled nature of off-target transposition, which increases the risk of genetic rearrangements in the genome of generated human iPSCs, transposase expression needs to be controlled (Bayart and Cohen-Haguenauer, 2013). These limitations of the transposon system have limited their practical use for iPSC reprogramming (Supplementary Table S1).

##### 3.2.1.2 DNA transfection

Another alternative to viral reprogramming is the delivery of pluripotency factors by transfection with a single multicistronic DNA vector that is capable of integrating into the host cell genome and reprogramming it, after which the exogenous reprogramming factors flanked by loxP sites can be completely removed from iPSCs by the subsequent expression of Cre recombinase (Kaji et al., 2009). This method was used to reprogram mouse fibroblasts; however, the low transfection efficiency and ambiguous assessment of the reprogramming efficiency, as well as the risk of reactivation of exogenous factors, random localization of integration, and residual fragments after Cre-mediated transgene removal, raise concerns about the application of this method. The same authors proposed combining this approach with PB transposons for the generation of human iPSCs; these transposons are removed from the integration site without the residual changes in the original DNA sequence and also promote more efficient stable expression.

#### 3.2.2 Non-integrative reprogramming methods

##### 3.2.2.1 Episomal vectors

As an alternative to viral delivery, reprogramming methods based on the delivery of episomal vectors have been developed. Episomes, which include plasmids and minicircles, are extrachromosomal DNA molecules that can autonomously replicate in cells and can be used for direct and transient transfection of pluripotency factors into somatic cells. Despite its simplicity, this method requires repeated transfections, since the duration of transgene expression from the plasmid is limited due to the gradual elimination of the episomes with each cell division (Bailly et al., 2022).

To circumvent the need for repeated transfections and to solve the problem of episome elimination during cell division, episomal vectors based on the oriP/Epstein-Barr nuclear antigen-1 (oriP/EBNA1) were developed (Yu et al., 2009). These vectors autonomously replicate as extrachromosomal elements, are maintained as stable episomes under the control of a selective inducer, and can be eliminated upon its removal (Yates et al., 1984; 1985). However, even with the use of replicating vectors, the efficiency of cell reprogramming remains low, as with other non-integrative systems (Yu et al., 2009).

The advantages of the episomal method include the diversity of cell types that can be successfully reprogrammed (e.g., skin fibroblasts, blood cells, mesenchymal stem cells, and urinary tract cells), simplicity and relatively low cost, and the availability of clinical-grade episomal reprogramming protocols (Karami et al., 2023; Schlaeger, 2018). However, the reprogramming efficiency using the episomal method remains quite low, and only a third of the resulting iPSCs are devoid of vector DNA; therefore, it is impossible to completely exclude the risk of genomic integration (Karami et al., 2023; Sridhar et al., 2016). Okita and colleagues improved the efficiency of the method by using three episomal plasmid vectors with five reprogramming factors (OSKML) and an additional hairpin RNA against TP53 to reprogram human dermal fibroblast lines and two dental pulp cell lines (Okita et al., 2011). However, TP53 knockout raises safety concerns as it may lead to genomic instability (Bailly et al., 2022; Marión et al., 2009). On the other hand, minicircle DNA vectors, unlike conventional plasmids, contain only the eukaryotic promoter and the transgene of interest and, therefore, allow for a reduction in the size of the reprogramming episomes. Compared with standard plasmid DNA, minicircle DNA vectors provide higher transfection efficiency and longer expression due to reduced silencing mechanisms (Bayart and Cohen-Haguenauer, 2013). Using this strategy, Jia et al. [10.1038/nmeth.1426] and Narsinh and colleagues (Narsinh et al., 2011) achieved the reprogramming of human adipose-derived stem cells with the OSKM combination of TFs with higher efficiency than plasmids (Bailly et al., 2022).

##### 3.2.2.2 Protein delivery of pluripotency factors

Delivery of pluripotency factors as proteins allows for induction of reprogramming without introduction of exogenous genetic material into donor cells. Zhou and colleagues reported the first successful recombinant protein-mediated reprogramming in 2009 using mouse fibroblasts (Zhou and Freed, 2009). Also in 2009, Kim and colleagues reprogrammed human fibroblasts using extracts from HEK293 cell lines. Each line expressed one of four OSKM TFs (Bailly et al., 2022; Kim et al., 2009), which were fused to the poly-arginine protein transduction domain (11R). After 6 weeks of regular exposure to protein extracts, several iPS colonies were isolated (Bayart and Cohen-Haguenauer, 2013; Kim et al., 2009). Although the method of reprogramming based on protein delivery of pluripotency allows generating iPSC lines completely devoid of exogenous DNA with a minimal risk of insertional mutagenesis, its low efficiency makes it less attractive compared to other methods (Bailly et al., 2022).

##### 3.2.2.3 Reprogramming with mRNA

Another reprogramming method relies on the delivery of synthetic mRNA encoding pluripotency factors. The main advantage of using mRNA compared to plasmid DNA is the fact that it only needs to enter the cell cytoplasm to initiate protein translation (Hayashi et al., 2010). Reprogramming with mRNA is considered safe as it is not possible for RNA to integrate into the host cell genome. It is also the most effective method compared to other non-viral, non-integrating delivery systems (Bailly et al., 2022). The disadvantages of using mRNA are the low stability of mRNA in the cytoplasm and its rapid degradation, which leads to a significant decrease in the expression of the delivered pluripotency factors and, as a consequence, the low efficiency of reprogramming (Cherkashova et al., 2020). Another significant limitation of the method is that synthetic mRNAs are capable of activating the innate immune system, which suppresses protein translation and triggers a cascade of cytotoxic and cytostatic reactions preventing reprogramming (Warren and Lin, 2019). While Plews and colleagues (Plews et al., 2010) in 2010 were the first to show that *in vitro* transcribed mRNAs of pluripotent factors lead to increased expression of endogenous TFs, the results of their work, unfortunately, did not lead to complete reprogramming. Several months later, Yakubov and colleagues (Yakubov et al., 2010) successfully reprogrammed human fibroblasts by performing five sequential transfections over several days using four *in vitro* transcribed mRNAs. Various modifications of the mRNA platform, including optimization of the 5′and 3′UTRs, the polyA tail, a synthetic cap analog, and the incorporation of modified uridine analogs, allowed for a significant increase in the efficiency of RNA translation and the subsequent reprogramming (Bayart and Cohen-Haguenauer, 2013). Interestingly, reprogramming could be achieved by a single transfection of self-replicating RNA (saRNA) containing alphavirus nonstructural gene sequences, allowing it to replicate inside the cell (Yoshioka et al., 2013). Steinle and colleagues (Steinle et al., 2019) showed that despite the fact that saRNA is considered more reactogenic due to the initiation of the replication process, saRNA-based reprogramming is more efficient and practical than mRNA-based reprogramming.

Significant progress has also been made in the past few years in adapting mRNA protocols and scaling up its production for iPSC-based therapies. In recent years, highly automated mRNA-based iPSC production lines have been implemented (Paull et al., 2015), GMP-compliant protocols for mRNA reprogramming and iPSC expansion have been described (Durruthy-Durruthy et al., 2014; Ni et al., 2016), and dedicated iPSC production facilities using these methods have been announced (Warren and Lin C., 2019).

##### 3.2.2.4 The role of microRNAs in the induction of pluripotency

MicroRNAs are able to regulate the amount of mRNA using the RNA interference mechanism and play a crucial role in cell reprogramming (Bailly et al., 2022). Some of the most well-known microRNAs are miR-302a, miR-302b, miR-302c, miR-302d, and miR-367, which are part of the miR-302-367 cluster and regulate the expression of more than 400 human genes (Rahimi et al., 2021). MiR-302-367 has been shown to downregulate stem cell differentiation-promoting genes, support somatic cell reprogramming (Hu et al., 2013; Ying et al., 2018), and improve male germline stem cell health (Zhu et al., 2018). Ectopic miR-302 expression can mediate stem cell reprogramming independent of the delivery of exogenous pluripotency factors such as Oct4, Sox2, Klf4, and c-Myc (Anokye-Danso et al., 2011; Miyoshi et al., 2011). Interestingly, these TFs bind to the miR-302 promoter region and regulate the expression of mouse miR-302 (Tian et al., 2011), and miR-302 expression levels have been reported to correlate with Oct4 expression levels (Hu et al., 2013).

The first evidence that somatic cells can be reprogrammed solely by microRNA expression was obtained in 2008 (Lin et al., 2008). A retroviral miR-302-367 microRNA expression system (Lin et al., 2008) was used to reprogram human cancer cells into ESC-like PSCs. Later, a similar result was obtained using human hair follicle cells and a new inducible expression vector, pTet-On-tTS-miR302, delivered to the cells by electroporation (Lin et al., 2011). Anoki-Danso and colleagues used the miR-302-367 cluster but with an LV delivery system to reprogram human fibroblasts, and the reprogramming efficiency was higher than when only using OSKM factors (Anokye-Danso et al., 2011). miRNAs can successfully reprogram somatic cells without a delivery system that integrates into the cell genome (Miyoshi et al., 2011). For example, transfection of miR-200c in combination with miRNAs from the miR-302-367 and miR-369 clusters ensured successful reprogramming of human dermal fibroblasts and human adipose tissue stromal cells (Miyoshi et al., 2011). The use of miRNAs for reprogramming somatic cells has a number of advantages. First, due to their small size, miRNAs are easier to transfect than mRNA or other reprogramming vectors. Moreover, the use of miRNAs is safer as no potential oncogenes are delivered to the cells. For example, c-Myc induces the expression of miR-141, miR-200, and miR-429, which block the differentiation of embryonic stem cells; delivery of these miRNAs eliminates the need for c-Myc (Cherkashova et al., 2020; Yang et al., 2011). Deng and colleagues reported the use of miRNAs 302-367 in place of Klf4 and c-Myc in the OSKM combination (Deng et al., 2015). Finally, their role as reprogramming enhancers allows microRNAs to increase reprogramming efficiency and/or reduce the number of transfections required when used in combination with mRNA delivery of pluripotency factor sequences (Bailly et al., 2022).

### 3.3 Non-viral delivery of transcription factors

In the case of viral reprogramming, pluripotency factor coding sequences are delivered by transduction with recombinant viral particles. This traditional method using, for example, LV vectors, demonstrates high efficiency and a low level of toxicity to cells (Cao et al., 2009). Non-viral reprogramming methods vary and include transfection and electroporation, among others. The most common chemical method is lipofection–liposome-based transfection. By means of electrostatic interactions, negatively charged nucleic acids bind to cationic lipids to form lipoplexes capable of penetrating the transfected cell by endocytosis or fusion with the membrane due to the presence of a phospholipid bilayer. This method is suitable for reprogramming using any type of nucleic acids: linear DNA, RNA, episomes (plasmids and minicircles), and transposons. Among the commercial liposomal reagents are various versions of Lipofectamine (Lipofectamine® 2000, Lipofectamine® 3000, Lipofectamine® RNAiMAX), as well as lipid-based Stemfect for RNA. Liposomal magnetofection is a variation of the method, which allows concentrating lipoplexes associated with magnetic nanoparticles on the surface of the transfected cells (Mykhaylyk et al., 2010). Other chemical transfection reagents include cationic polymers, which form polyplexes absorbed by the cell via endocytosis (DEAE-dextran, PEI), non-liposomal FuGENE, and some others.

Another common method of delivering nucleic acids into cells is electroporation, a physical method that allows direct (without binding to any reagent) delivery of nucleic acids into the cells due to a temporary increase in the permeability of cell membranes after short-term exposure to a high-voltage current. This method demonstrates higher efficiency than lipofection (Cao et al., 2009) and is suitable for reprogramming of difficult-to-transfect cells, such as primary and stem cells (Chong et al., 2021), but is labor-intensive and leads to a high frequency of cell death as the efficiency of nucleic acid delivery and cell viability depends on the voltage and duration of the electroporation process (Chong et al., 2021). Another widely used method, nucleofection, is an Amaxa Nucleofector-based electroporation, which uses a cell type-specific combination of electrical parameters and solutions. This method demonstrates significantly higher efficiency compared to electroporation and lipofection and also provides higher survival than electroporation (Cao et al., 2009) and is carried out by various commercial kits (Nucleofector™, Neon®, etc.). Electroporation-based methods are suitable for reprogramming using episomes, transposons, linear DNA, and RNA. Protein delivery of pluripotency factors into cells is possible using cell permeabilization agents, which temporarily create holes in the cell membrane, or using cell-penetrating peptides (Seo et al., 2017). Delivery of microRNA is possible using both traditional viral methods and non-viral liposomal and polymeric systems or exosomes (Dasgupta and Chatterjee, 2021). Other less commonly used physical delivery methods include sonoporation, magnetoporation, gene microinjection, and laser irradiation (Chong et al., 2021).

An emerging promising method is the delivery of nucleic acids using self-assembling virus-like particles (VLPs), which lack their infectious and replicative abilities. This method is of interest due to the safety and biocompatibility of VLPs, the possibility of producing their large quantities in recombinant systems, as well as the ease of modification of external/internal surfaces that improve binding and targeting to a specific cell type (Ikwuagwu and Tullman-Ercek, 2022), and, theoretically, can be used to deliver pluripotency factors.

## 4 Barriers to cell reprogramming and subsequent application of iPSCs

**FIGURE 2:**
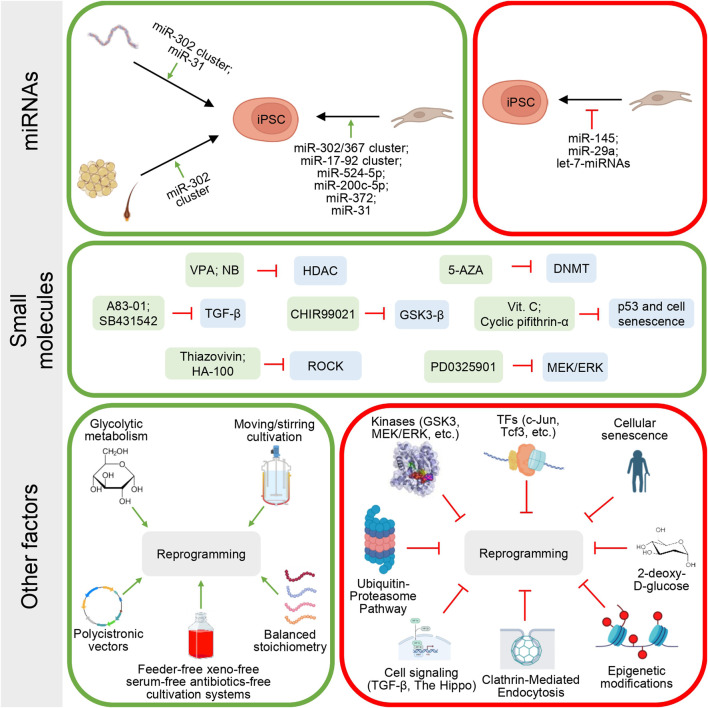
Barriers to cell reprogramming and approaches used to overcome them. Various approaches are used to increase the reprogramming efficiency.

Activation of specific signaling pathways during reprogramming can interfere with the induction and maintenance of cell pluripotency (Figure 2). At early stages, the TGF-β signaling pathway blocks cellular reprogramming by preventing mesothelial-to-epithelial transition (MET) and promoting pro-epithelial-to-mesenchymal transition (EMT) signals. At later stages of reprogramming, TGF-β activation interferes with achieving terminal pluripotency by promoting cell arrest in an intermediate, partially reprogrammed state (Haridhasapavalan et al., 2020). The Hippo signaling pathway suppresses activation of the Wnt/β-catenin pathway (Heallen et al., 2011; Varelas et al., 2010), which is involved in the maintenance of pluripotency in mammalian stem cells (Hao et al., 2006; Sato et al., 2004; Xu et al., 2016), as well as the induction of pluripotency in somatic cells (Haridhasapavalan et al., 2020; Lluis et al., 2008; Marson et al., 2008). Activation of signaling pathways associated with various protein kinases was identified as a barrier to cellular reprogramming. These protein kinases include glycogen kinase 3 (GSK3), mitogen-activated protein kinase/extracellular signal-regulated kinase 1/2 (MEK/ERK), Rho-associated protein kinase (Lai et al., 2010), protein kinase C (Lin et al., 2018), and Src family tyrosine kinase (Staerk et al., 2011). Inhibition of these signaling pathways is important for increasing the efficiency of reprogramming.

Some specific transcription factors, such as c-Jun, Tcf3, Bright/ARID3A, GATA4, Zfp281, or Patz1, can also reduce the efficiency or even block the reprogramming process. Their expression can lead to the suppression of pluripotency-associated genes, inhibition of the MET transition, and/or a number of other processes (Haridhasapavalan et al., 2020). In addition to the activation of signaling pathways, certain cell characteristics also affect the reprogramming success. One of the key factors is cellular senescence, which presents a barrier to reprogramming. Cell senescence leads to oxidative stress, DNA damage, telomere shortening, and suppression of the Ink4a/Arf locus activation through chromatin remodeling, halting cell proliferation and division (Collado et al., 2007; Utikal et al., 2009). The generation of oxidative stress observed in senescent cells is one of the leading causes of DNA damage, which triggers the induction of p53 and its target p21 during reprogramming (Banito et al., 2009; Hong et al., 2009; Jiang et al., 2013; Kawamura et al., 2009), and results in p53-p21-dependent cell cycle arrest or apoptosis (Marión et al., 2009). Other aging regulators, such as activation of the Ink4a/Arf locus (Banito et al., 2009; Li W. et al., 2009; Utikal et al., 2009) and its two components, p19Arf (Li H. et al., 2009; Utikal et al., 2009) and p16Ink4a (Banito et al., 2009; Li W. et al., 2009), are involved in repression of reprogramming (Haridhasapavalan et al., 2020).

Some epigenetic modifications can become an obstacle to the reprogramming of somatic cells. Global DNA methylation catalyzed by DNA methyltransferases prevents the binding of transcription factors to the promoter and other regulatory regions of pluripotency genes and gene induction during cellular reprogramming (Haridhasapavalan et al., 2020; Stadtfeld and Hochedlinger, 2010). Histone (H3K4, H3K9, H3K27, H3K36, H3K79, etc.) methylation suppresses the expression of the most important pluripotency genes and prevents cellular reprogramming. In particular, histone methyltransferase G9a causes trimethylation at lysine 9 of histone H3 (H3K9me3) to form heterochromatin by recruiting heterochromatin protein 1 (Epsztejn-Litman et al., 2008; Feldman et al., 2006), which makes regulatory regions of DNA less accessible for transcription factor binding (Haridhasapavalan et al., 2020). Deacetylation of histones by histone deacetylase enzymes (HDAC) also enhances heterochromatization processes, complicating the initiation of gene transcription (Haridhasapavalan et al., 2020; Huynh et al., 2017; Seto and Yoshida 2014).

Clathrin-mediated endocytosis also presents a barrier to cellular reprogramming as it prevents the MET transition by activating TGF-β signaling and components of the ubiquitin-proteasome pathway. This promotes the degradation of pluripotency-associated genes, as well as some others (Haridhasapavalan et al., 2020).

Some miRNAs have also been identified as barriers to the generation of human iPSCs. For example, miR-145 (Barta et al., 2016) and miR-29a (Hysolli et al., 2016) are expressed at high levels in the cells being reprogrammed (fibroblasts) and at low levels in pluripotent cells. They stimulate the expression of genes promoting differentiation (Let-7 family) (Worringer et al., 2014) or, conversely, may inhibit the Oct4, Sox2, and Klf4 genes (miR-145) (Borgohain et al., 2019).

## 5 Methods for improving the efficiency of reprogramming and maintaining pluripotency

In addition to optimizing the delivery, composition, and ratio of delivered transcription factors that induce pluripotency, the search for other universal methods for increasing the efficiency of cell reprogramming and maintaining pluripotency continues.

### 5.1 Polycistronic cassettes and the optimal ratio of pluripotency factors

Polycistronic cassettes encoding several pluripotency factors at once are considered more efficient for reprogramming (Carey et al., 2009) as they can be delivered by a single vector. This allows a guaranteed ectopic expression of all transcription factors at an equimolar ratio in the targeted cells and significantly reduces the vector load on the cell. Within a single polycistronic cassette, it is possible to encode pluripotency factors in such a way that the optimal stoichiometry of their expression levels is post-translationally maintained (Haridhasapavalan et al., 2020).

While the combination of certain factors is important, it is not the only key factor for the successful reprogramming. Induction of pluripotency can be equally affected by the levels of expression of the exogenous transcription factors delivered to the cells or/and by their endogenous levels, if present, depending on the cells used for reprogramming. Out of the four transcription factors (c-Myc, Oct3/4, Sox2, and Klf4), it was the expression of c-Myc that resulted in the most prominent ESC-like expression pattern in reprogrammed fibroblasts (Sridharan et al., 2009). While Oct3/4 and Sox2 are considered key transcriptional factors that inhibit the expression of genes associated with differentiation (Gillis et al., 2011), they cannot exert their function on methylated target sequences unless c-Myc fulfills its mission first (Takahashi et al., 2007). The role of Oct3/4 is supported by the finding that its absence results in failure of iPSC colony generation. This transcription factor also needs to interact with Sox2 and Klf4 in order to activate ESC-specific genes partially silenced in reprogrammed cells (Shi and Jin, 2010; Sterneckert et al., 2012). Sox2 plays a crucial role but in a dose-dependent manner and reverses the silenced epigenetic signature of differentiated cells to a pluripotent ESC-like state. The levels of expression and the ratio of Sox2 and Oct3/4 affect the reprogramming efficiency and quality of iPSCs colonies. For example, higher levels of Oct3/4 as compared to other transcription factors were shown to increase the reprogramming efficiency, which was negatively affected by both its decrease and the higher levels of Sox2. Interestingly, the decrease in Sox2 levels expressed in combination with Oct3/4, Sox2, and Klf4 increased the efficiency of generating partially reprogrammed iPSCs (Gillis et al., 2011; Yamaguchi et al., 2011; Matsuoka et al., 2012). Low Sox2 expression was also linked with the reduced expression of ectoderm and mesoderm marker genes, indicating the defects in ectodermal and mesodermal lineage differentiation. Either a decrease in Sox2 on its own or in combination with the increase in Oct3/4 improved the reprogramming of mouse somatic cells (Carey et al., 2011; Rizzino, 2013). The important role of Oct3/4 was additionally confirmed by the onset of apoptosis in embryonic cells with Oct3/4 knock-out (Yamaguchi et al., 2011; Matsuoka et al., 2012). Overexpression of Lin28A, another transcription factor, with Oct3/4, Sox2, and Nanog helped the reprogramming of human somatic fibroblasts into self-renewing iPSCs (Yu et al., 2007; Matsuoka et al., 2012). Upon induction of Sox2 expression, Lin28A is used as one of the earliest markers of somatic cell reprogramming, the absence of which affects the number of iPSC colonies (Yu et al., 2007; Shi and Jin, 2010; Gillis et al., 2011; Shyh-Chang et al., 2013; Wang et al., 2019). The cumulative effect of Sox2, Oct3/4, and Klf4 is required for the complete epigenetic changes to take place and result in the generation of fully reprogrammed cells. Interestingly, Klf4 can potentially be replaced not only by similar factors Klf2 and Klf5 but also by Esrrb (a transcription factor that binds a canonical ESRRB recognition ERRE) when co-transduced with either Oct3/4, Sox2, and c-Myc or Oct3/4 and Sox2 to reprogram mouse embryonic fibroblasts (Nakagawa et al., 2008; Feng et al., 2009a). Esrrb acts as a transcriptional activator of Klf4, Oct4, Sox2, and Nanog (Van Den Berg et al., 2008; Zhang et al., 2008). It is also noteworthy that Sox2, Nanog, and Esrrb are physically associated with Oct4 (Wang et al., 2006; Liang et al., 2008).

### 5.2 Small molecules

In 2011, the developed cocktail of six small molecules increased the efficiency of episomal reprogramming of fibroblasts by 70 times (Yu et al., 2011). Among these molecules are CHIR99021 (glycogen synthase kinase GSK3β inhibitor), PD0325901 (mitogen-activated protein kinase MEK inhibitor), human LIF (cytokine self-renewal inhibitory factor in leukemia), A-83-01 (TGF-β/activin/nodular receptor inhibitor), bFGF (fibroblast growth factor), and HA-100 (Rho kinase inhibitor) (Liu G. et al., 2020). Later in 2016, Di Li and colleagues (Li et al., 2016) proposed another combination of small molecules to enhance the reprogramming efficiency of human urinary iPSCs. This cocktail, including cyclic pifithrin-a (a P53 inhibitor), A-83–01, CHIR99021, thiazovivine (a Rho kinase inhibitor), sodium butyrate (NaB, a histone deacetylase inhibitor), and PD0325901, significantly increased reprogramming efficiency (Li et al., 2016). The combination of PD0325901, a MEK inhibitor, and LIF enhances reprogramming efficiency (Silva et al., 2008). PD0325901 enhances iPSC generation from neural progenitor cells, promoting pluripotency. It also selectively binds and inhibits MEK, which can induce inhibition of phosphorylation and activation of MAPK/ERK and thus inhibit tumor cell proliferation (Lin et al., 2009; Yu et al., 2011; Zhu et al., 2010). In addition, PD0325901 promotes the growth of iPSCs while suppressing the growth of non-iPSCs (Shi Y. et al., 2008b). A-83-01 promotes the reprogramming of human epidermal keratinocytes via inhibition of TGF-β (Yu et al., 2011; Zhu et al., 2010). High concentrations of bFGF support the growth of ESCs and human ESC-like iPSC colonies through several pathways other than MEK (Yu et al., 2011). Cyclic pifithrin-α suppresses P53, thereby significantly enhancing the reprogramming capacity of human somatic cells (Hong et al., 2009). HA-100 and thiazovivine, ROCK inhibitors, both significantly enhance reprogramming efficiency in the presence of PD, Chir, A-83-01, and hLIF (Yu et al., 2011). Sodium butyrate stimulates miR302/367 clusters, histone H3 acetylation, DNA demethylation, and expression of endogenous genes associated with pluripotency (Mali et al., 2010; Zhang and Wu, 2013). Reprogramming of somatic cells is possible not only with the help of exogenous transcription factors but also with the help of exclusively small molecules that modulate molecular pathways that are not specific for pluripotency (they do not include direct activation of classical reprogramming factors). This method of generating CiPSCs (chemically induced pluripotent stem cells) is considered promising since small molecules are able to penetrate cells, are non-immunogenic, are more economical, and are easier to synthesize, store, and standardize than traditional factors. Moreover, their effects on inhibition and activation of specific protein function are often reversible and can be fine-tuned by adjusting concentrations (Hou et al., 2013).

For some time it was assumed that cell reprogramming using chemical compounds alone was impossible. This was associated with a significant reduction in the number of generated iPSC clones with chemical replacement of the transcription factor, as well as the risk of introducing genetic or epigenetic abnormalities into the resulting iPSCs, since many of the described compounds are modulators of DNA and chromatin modifications (Stadtfeld and Hochedlinger, 2010). However, in 2013, Hou and colleagues showed (Hou et al., 2013) that induction of iPSCs from mouse fibroblasts is possible with a cocktail of seven small molecules: valproic acid (VPA, a histone deacetylase inhibitor), CHIR99021 (a glycogen synthase kinase inhibitor, GSK3β), E-616452 (RepSox, a TGF-β receptor inhibitor), tranylcypromine (a monoamine oxidase inhibitor), forskolin (an adenylyl cyclase activator), 3-deazaneplanocin A (DZNep, a histone methyltransferase inhibitor), and TTNPB (a retinoid pathway activator). Further, Zhao and colleagues in 2015 (Zhao et al., 2015) achieved a 1000-fold increase in the efficiency of the previous protocol by adding four new small molecules: AM580, EPZ004777, SGC0946, and AZA (Liu A. et al., 2020). Some of the molecules are known to be able to replace individual transcription factors or even their combinations (Stadtfeld and Hochedlinger, 2010) and allow for full cell reprogramming in the presence of the remaining necessary pluripotency factors.

Chemical screening by Ichida and colleagues (Ichida et al., 2009) identified a RepSox molecule (E616452, a TGF-β inhibitor) capable of replacing Sox2. Kenpollon (a GSK3β inhibitor) was found to functionally replace Klf4 in the presence of OSM (Lyssiotis et al., 2009), and a combination of BIX01294 and Bayk8644 or BIX01294 and RG108 mediated reprogramming of mouse fibroblasts in the presence of OK. Li and colleagues (Li H. et al., 2009; Shi Y. et al., 2008a) also reported successful OK reprogramming of human somatic cells using CHIR99021 and tranylcypromine. It was shown that VPA can promote OS-induced reprogramming of human fibroblasts (Huangfu et al., 2008a; Ma et al., 2017). Bromodeoxyuridine (BrdU), a thymidine analogue, was found to be able to replace Oct4 and generate iPSCs in the presence of SKM (Bailly et al., 2022; Long et al., 2015). Subsequently, several groups focused solely on Oct-dependent reprogramming using various small molecules (Ma et al., 2017). The combination of AMI-5 and A83-01 with Oct4 successfully reprogrammed mouse fibroblasts (Yuan et al., 2011). Other studies identified a specific chemical combination consisting of VPA, CHIR99021, E616452, and tranylcypromine that was sufficient to reprogram mouse fibroblasts into iPSCs using Oct4 alone (Li et al., 2011; Ma et al., 2017). Moreover, Forskolin, an activator of cAMP signaling, as well as the serotonin 5-HT receptor agonists D4476 and 2-methyl-5-hydroxytryptamine (2-Me-5-HT), have been shown to independently replace Oct4 (Hou et al., 2013).

Despite the attractiveness of using small molecules for reprogramming due to the simplicity and potential scalability of the method (Cerneckis et al., 2024; Guan et al., 2022; Kim et al., 2020; Liuyang et al., 2023), it has not yet been possible to obtain induced human iPSCs using small molecules alone. This is due to significant differences between mice and humans in epigenetic memory and pluripotency signaling pathways (Kim et al., 2020; Papp and Plath, 2013; Scesa et al., 2021; Zhong et al., 2022). Chemical reprogramming of human cells requires the selection and optimization of new combinations and concentrations of small molecules, and a detailed analysis and comparison of signaling pathways in human and mouse cells can help with this. Thus, the use of small molecules together with pluripotency factors helps to increase the reprogramming efficiency and can be used to obtain iPSCs (Liu G. et al., 2020).

### 5.3 miRNAs

Several other miRNAs, in addition to the entire miR-302-367 cluster (see Section 1, Reprogramming Methods), have been identified as inducers of the reprogramming process in human cells. Their mechanism of action involves suppression of the EMT transition (by inhibiting TGF-β signals) or stimulation of the MET transition (miR-302b and miR-372 (Subramanyam et al., 2011), miR-524-5p (Nguyen et al., 2017)), stimulation of the transition from mitochondrial respiration to glycolytic metabolism (miR-31 (Lee et al., 2016), miR-200c-5p (Cha et al., 2017)), enhancement of global demethylation (miR-302 (Lin et al., 2011)), as well as targeted inhibitory action on certain genes (miR-17-92 cluster (He et al., 2014)) or transcription factors (miR-302 cluster (without miR-367) (Borgohain et al., 2019; S; Hu et al., 2013).

### 5.4 Cultivation conditions

The efficiency of reprogramming and maintenance of the pluripotent status of cells are mainly influenced by the culturing conditions. The traditional protocol involves culturing iPSCs on a monolayer of feeder cells, usually primary, mitotically inactivated fibroblasts. These feeder cells secrete important growth factors, extracellular matrix components, and cytokines into the nutrient medium, which support the growth and proliferation of pluripotent cells (Dakhore et al., 2018; Sams and Powers, 2013; Yao et al., 2006). This culturing method is generally accepted but has a number of disadvantages: it is labor-intensive and difficult to scale up (Sams and Powers, 2013). Moreover, feeder cells can potentially become a source of pathogens and *mycoplasma* contamination (Dakhore et al., 2018; Mannello and Tonti, 2007; Sams and Powers, 2013) and can also complicate further characterization of the iPSC population (Castro-Viñuelas et al., 2021; Skottman and Hovatta, 2006). Currently, the development of protocols for iPSC cultivation in the absence of a feeder is of paramount importance, as they not only ensure the reliability, reproducibility, sustainability, efficiency, and safety of the process but also accelerate the process (due to the absence of the stage of preparation of feeder layer cells), facilitate scaling, and enable high-throughput screening (Healy and Ruban, 2015). Nutrient media for iPSC cultivation should also be xeno- and serum-free and should not contain other components capable of inducing non-target differentiation of iPSCs (Jung et al., 2012). Traditional methods involve culturing iPSCs in a static environment. However, stirred microcarrier cultures are currently gaining popularity, allowing the production of high concentrations of iPSCs, as well as scaling up further expansion and differentiation of cells in bioreactors. In a mobile system, medium circulation is believed to provide uniform nutrition to the cells, and physical stimulation can promote growth (Liu A. et al., 2020). Long-term maintenance of iPSC cultures is accomplished using scalable, stable, and cost-effective flasks with various biological (e.g., Matrigel, fibronectin, vitronectin, or laminin (Healy and Ruban, 2015), CELLstart™) or synthetic (e.g., polyacrylamide-co-propargyl acrylamide) matrices. Animal-derived materials can also potentially be used as matrices (Liu G. et al., 2020).

It is also worth mentioning that antibiotics are not used in laboratory practice for culturing stable iPSC lines: this prevents masking contamination by bacteria or fungi, thereby allowing for their rapid detection. The presence of viral contamination can be determined by the cytopathic effect, and *mycoplasma* infection can be determined by various laboratory tests (Healy and Ruban, 2015).

### 5.5 Stimulation of glycolysis

At the initial stages of reprogramming, a transition from a predominantly oxidative to a predominantly glycolytic metabolic phenotype occurs, reminiscent of ESC phenotype (Varum et al., 2011). Maintaining a physiological (5%) oxygen concentration, adding D-fructose-6-phosphate (F6P) (an intermediate product of glycolysis) to the medium, or increasing the level of HIF1α, a transcription factor that activates glycolytic genes, stimulates glycolysis and promotes efficient reprogramming. In turn, 2-deoxy-D-glucose (2-DG), an inhibitor of glycolysis, reduces the conversion of glucose to lactate and, accordingly, the efficiency of reprogramming (Spyrou et al., 2019). It has also been demonstrated that physiological hypoxia (3%–5% O2), characteristic of the bone marrow niche, maintains the expression of pluripotency markers and prevents spontaneous differentiation of iPSCs (Nit et al., 2021). At 5% oxygen, the reprogramming efficiency increases approximately 5-fold for mouse cells and 3-fold for human cells (Malik and Rao, 2013; Yoshida Y. et al., 2009), compared to normoxic conditions. However, long-term exposure to hypoxic conditions (up to 25 days) can reduce the efficiency of reprogramming and disrupt colony morphology (Iida et al., 2013; Nit et al., 2021).

## 6 Reprogramming features depending on the cell type

Certain reprogramming parameters may vary depending on the cell type used to obtain iPSCs. As described earlier, some cell types do not require delivery of certain reprogramming factors, since they are expressed endogenously at high levels. The somatic cell type also affects the efficiency and kinetics of reprogramming, although it is not always possible to directly compare these parameters due to the different delivery methods used in these studies (Brouwer et al., 2016). Cells undergoing reprogramming should meet a number of requirements. First, the cells should be easily accessible for collection using a minimally invasive procedure; second, the cells should be well cultured and highly proliferative to obtain a large pool of cells free of critical somatic mutations and chromosomal aberrations; and third, the cells should have the ability to generate iPSCs with high efficiency (Bailly et al., 2022).

### 6.1 Skin cells

Historically, fibroblasts were the first cell type to undergo reprogramming in the pilot studies of Yamanaka and Takahashi: first, using the “Yamanaka cocktail,” the research group obtained iPSCs from mouse fibroblasts (Takahashi and Yamanaka, 2006), after which the result was successfully reproduced with human fibroblasts (Takahashi et al., 2007). In addition to mouse and human fibroblasts, rat fibroblasts (Liao et al., 2008) and rhesus macaque fibroblasts (Bailly et al., 2022; Liu C. et al., 2018) were also reprogrammed in a number of studies.

Dermal fibroblasts have traditionally been obtained by skin punch biopsy, which, although a well-established technique, remains an invasive procedure. In addition, successful reprogramming requires maintaining multiple cell passages, which is labor-intensive (Staerk et al., 2010). However, fibroblast isolation and culture protocols have proven themselves to be quite effective, and fibroblasts remain a widely used cell source for reprogramming (Bailly et al., 2022).

Other dermal cells, such as melanocytes and keratinocytes, can also be obtained by skin biopsy. Keratinocytes have been shown to be reprogrammed more quickly and efficiently than fibroblasts and are a much more accessible source, since in addition to punch biopsy, they can be isolated from hair (Aasen et al., 2008; Aasen and Belmonte, 2010; Bailly et al., 2022; Piao et al., 2014). A study by Utikal and colleagues (Utikal et al., 2009) showed that human and mouse melanocytes gave rise to iPSCs with higher efficiency than fibroblasts or keratinocytes. This is due to high endogenous expression of the Sox2 factor, which allows reprogramming of these cells without its delivery (Utikal et al., 2009). A significant disadvantage of iPSCs derived from skin cells is the increased content of common mutations associated with exposure to ultraviolet radiation. In addition, iPSCs from fibroblasts exhibit genomic heterogeneity (Cerneckis et al., 2024).

### 6.2 Peripheral blood cells

Peripheral blood cells, such as CD34^+^ hematopoietic stem cells (HSCs) (Loh et al., 2009; Mack et al., 2011; Ye et al., 2009), blood mononuclear cells (MNCs) (Dowey et al., 2012), and T lymphocytes (Loh et al., 2009; Seki et al., 2012; 2010; Staerk et al., 2010), are widely used to obtain iPSCs. The first successful reprogramming of peripheral blood cells (T cells and myeloid cells) was performed by Staerk and colleagues in 2010 (Staerk et al., 2010). These cells, unlike fibroblasts, are easily accessible (samples can be stored frozen) and do not require intensive cell culture maintenance before experiments (Staerk et al., 2010), although in general, culturing blood cells remains a complex process (Bailly et al., 2022). iPSCs obtained from peripheral blood mononuclear cells have fewer mutations than iPSCs obtained from skin fibroblasts due to the lower exposure to ultraviolet light (Cerneckis et al., 2024). A limitation of blood cell reprogramming is their low susceptibility to transfection with cationic reagents, which is a serious obstacle to their reprogramming using lipotransfection systems. Electroporation can be used as an alternative method for delivering non-viral reprogramming systems (Rabinovich et al., 2009; Van Tendeloo et al., 2001); however, the side effects of electroporation on cell viability limit the possibility of its repeated use for several days in a row (Warren and Lin C., 2019). It has also been shown that terminally differentiated blood cells (B and T cells) are less amenable to reprogramming than HSCs (Bayart and Cohen-Haguenauer, 2013; Eminli et al., 2009).

### 6.3 Mesenchymal stem cells

Another option for reprogramming cells is mesenchymal stem cells (MSCs), which can be isolated from various surgical and biological waste materials. For example, such cells can be obtained from adipose tissue (Jia et al., 2010; Narsinh et al., 2011; Sun et al., 2009), dental tissue (Yan et al., 2010), umbilical cord blood (Haase et al., 2009; Meng et al., 2012; Okita et al., 2013; Yan et al., 2010), or even from urine (Xue et al., 2013; Zhou et al., 2012; 2011). However, most of these MSC sources are obtained through invasive surgery, which significantly complicates their utilization (Bailly et al., 2022). An exception are renal tubular cells, which can be obtained from urine (Liu A. et al., 2020), which distinguishes them from other types of MSCs for reprogramming. The first iPSCs from renal tubular cells were obtained in 2011 (Zhou et al., 2012; 2011), and in 2020, Bouma and colleagues published a protocol for reprogramming urine-derived cells using a commercial self-replicating RNA kit and single-step electroporation (Bouma et al., 2020), which is also suitable for human olfactory neurosphere-derived cells (Leeson et al., 2021). Similarly, the StemRNATM-SR Reprogramming Kit from Stemgent/Reprocell was used to generate iPSCs from cord blood- or peripheral blood-derived endothelial progenitor cells (Eminli et al., 2021; Gao et al., 2018; 2017).

### 6.4 Other cell types

In addition to the cells listed above, iPSCs have also been derived from other somatic cell populations, such as neural cells (Eminli et al., 2008; Kim J. B. et al., 2008), gastric and liver cells (Aoi et al., 2008), and pancreatic β-cells (Stadtfeld et al., 2008a). These cell types are less accessible, which significantly complicates their use as sources of iPSCs (Stadtfeld and Hochedlinger, 2010). However, it is worth noting that neural progenitor cells (NPCs), like melanocytes, do not require ectopic Sox2 expression for reprogramming due to their high endogenous Sox2 levels (Ellis et al., 2004; Utikal et al., 2009).

Supplementary Table S1 provides information on the expected reprogramming efficiency for different cell types, as well as possible ways to improve the efficiency and/or safety of a particular reprogramming method. The efficiency level is presented as a range of values in accordance with the published studies that reported reprogramming of human cells with either the traditional Yamanaka factor cocktail or various combinations of these factors. Blanks in the table indicate the absence of published studies using certain cell types. ND (not determined) corresponds to studies that did not indicate the efficiency level.

## 7 Analysis of the obtained iPSCs

An important stage in obtaining iPSCs is their analytical characterization using various approaches. Such analysis is necessary to identify the iPSC colony among other differentiated or partially reprogrammed iPSCs.

### 7.1 Morphological analysis

Specific morphological features characteristic of iPSC colonies and distinguishing them from differentiated cells include a high nucleus to cytoplasm ratio, the presence of noticeable protruding nucleoli, and the formation of round, flat, compact colonies with a clearly defined and smooth edge.

Evaluation of morphological characteristics is extremely important for maintaining the pluripotent state of cells, since the presence of incorrectly reprogrammed cells in the culture at the early stages after reprogramming leads to deviation of cells from the undifferentiated state. It is on the basis of morphological features that it is necessary to detect and remove unsuitable cells and retain only those cells that have been correctly reprogrammed. Cell quality assessment is performed by visual inspection, which is a traditional but labor-intensive method with a high level of subjective errors. Several non-invasive methods based on image analysis and machine learning technologies have been proposed to replace visual inspection, which classifies cells into several quality classes using both non-morphological features (such as brightness intensity distribution in cell images (Maddah et al., 2014; Tokunaga et al., 2014; Kato et al., 2016) and morphological features used during the culturing process (Wakui et al., 2022).

### 7.2 Analysis of pluripotency markers using antibodies

iPSCs are identified by the expression of certain highly expressed markers that relate to their pluripotent status. The most well-known pluripotency markers include the transcription factors Oct3/4, Sox2, and Nanog; the tumor rejection antigens TRA-1-60 and TRA-1-81; and the embryonic stage-specific antigens SSEA3 and SSEA4 (The International Stem Cell Initiative, 2007). It is worth mentioning that hPSCs are negative for SSEA1, a pluripotency marker of murine PSCs (Pera et al., 2000). In humans, SSEA1 is expressed during differentiation; therefore, hPSCs should be negative for SSEA1 (Rehakova et al., 2020).

Flow cytometry is widely used to detect various cellular markers, including those responsible for the pluripotent state. Surface markers (TRA-1-60, TRA-1-81, SSEA3, SSEA4) are easier to detect since the antigens are accessible to antibodies, while the detection of intracellular markers (Oct3/4, Sox2, Nanog) requires an additional fixation step (Rehakova et al., 2020).

Flow cytometry is also widely used to detect iPSC heterogeneity since it is reliable and easy to perform, and the results obtained are quantitative and comparable across laboratories. Although there is no consensus on the criteria for clinical use, Baghbaderani and colleagues proposed that over 70% of cells should be positive for SSEA4, Oct3/4, TRA-1-60, and TRA–1-81, and less than 5% of cells must be negative for CD34^+^ (Baghbaderani et al., 2015; Zhong et al., 2022). Other options for criteria were discussed by Rehakova and co-authors (2020). Another advantage of the flow cytometry method is its scalability using fluorescence cell barcoding (FCB) technology (Krutzik and Nolan, 2006; D’Antonio et al., 2017). Also, efficient isolation of cells from a population based on specific markers can be performed using fluorescence-activated cell sorting (FACS) and magnetic activation cell sorting (MACS) technologies (Zhong et al., 2022). However, despite all the advantages, a certain disadvantage of the method is that it does not provide an idea of the spatial expression of antigens on cells in a colony or in a cell monolayer (Healy and Ruban, 2015). Another method based on the identification of pluripotency markers is immunofluorescent analysis. The advantage of this method is that in addition to detecting the presence of an antigen, it is possible to assess its intracellular localization (Rehakova et al., 2020). However, this method is usually used after the appearance of colonies and, therefore, cannot be used at an early stage of the reprogramming process.

### 7.3 Alkaline phosphatase activity

In addition to the assessment of specific markers by flow cytometry and immunofluorescent analysis, identification of iPSCs is also possible using specific chemical staining with alkaline phosphatase (AP). This enzyme has high activity in PSCs (including undifferentiated ESCs, embryonic germ cells, and iPSCs) and is capable of hydrolyzing cellular phosphate under alkaline conditions (Zhong et al., 2022).

Unlike antibody-based assays, AP substrates can be used at an early stage of screening, and the presence and the number of AP-positive colonies serve as a primary indicator of reprogramming efficiency (Singh et al., 2012).

Traditional AP staining methods require cell fixation and also lead to the accumulation of toxic reagents or end products of degradation inside the cells, which negatively affects the iPSC morphology and subsequent cultivation (Singh et al., 2012). However, methods for intravital staining have been developed, for example, using a fluorogenic substrate that penetrates living cells (AP Live Stain). As a result of enzymatic cleavage, a bright green fluorescent product is formed, which then diffuses from the cell without accumulating and without leaving a significant chemical or biological trace behind (the signal inside the cells disappears 2 hours after treatment). Such intravital staining can be performed repeatedly throughout the entire process of iPSC expansion, facilitating real-time monitoring of the reprogrammed colonies without affecting the cell integrity. However, positive staining for AP activity in itself is not a specific marker for iPSC clones; the pluripotency of the selected colonies should be further confirmed by other methods.

### 7.4 Monitoring of genomic stability

Genomic instability may occur at any stage of iPSC production, causing mutations that may be a significant obstacle to subsequent clinical iPSC applications from the safety and efficacy point of view. At least three sources of genetic variations in iPSCs are currently distinguished: 1) pre-existing variations in parental somatic cells that may be manifested during the cloning procedure during iPSC generation, 2) reprogramming-induced mutations that occur during reprogramming, and 3) mutations that occur during long-term culturing of cells (Yoshihara et al., 2017).

Genomic stability of iPSCs can be monitored by karyotype analysis using Giemsa-banding. This method allows the detection of numerical (aneuploidy and polyploidy) or large structural chromosomal changes, including translocations and inversions (Yoshihara et al., 2017; Yunis, 1976); however, this method is expensive, labor-intensive, and also difficult for mass analysis of iPSCs (D’Antonio et al., 2017). To achieve higher resolution, hybridization-based technologies (aCGH) (Kallioniemi et al., 1992) and single nucleotide polymorphism (SNP) genotyping (Wang et al., 1998) have been developed to investigate mutations across the entire genome (Yoshihara et al., 2017). The digital karyotyping method using whole-genome SNP genotyping is particularly popular since this highly sensitive method allows the investigation of the genomic integrity of iPSC lines at different stages (D’Antonio et al., 2017). However, SNP genotyping cannot detect balanced translocations and inversions (Riegel, 2014; Yoshihara et al., 2017). Thus, for a more accurate assessment of mutations in iPSC cells, it is necessary to combine SNP genotyping and karyotype analysis to obtain complete information on all possible chromosomal aberrations (Rehakova et al., 2020).

In addition to karyotyping, short tandem repeat (STR) analysis can establish the authenticity and genetic stability of iPSCs. Using this method, an unambiguous identification of a specific iPSC line is carried out: the STR profile of iPSCs is established at early passages and must strictly correspond to the profile of the cell donor (Rehakova et al., 2020).

Modern methods such as next-generation sequencing (NGS), whole-genome sequencing (WGS), and whole-exome sequencing (WES) allow the detection of high-resolution genome-wide genetic variations (Metzker, 2010) and low-frequency variations that cannot be identified by other methods (Pagnamenta et al., 2012; Yoshihara et al., 2017). However, due to the complexity of sample preparation and data processing, as well as the high cost of analysis, sequencing methods are less commonly used to assess the genetic profile of iPSCs.

Thus, monitoring and maintaining the genomic stability of iPSCs is critical for the efficacy and safety of subsequent clinical use.

### 7.5 Pluripotency-associated gene expression analysis

As described previously (see factors and mechanism of pluripotency), complete reprogramming of somatic cells occurs only when the expression of endogenous genes associated with pluripotency (Oct4, Sox2, Klf4, Nanog, Lin28, hTERT, REX1, SALL4, DPPA2, DPPA4, GDF3, cMyc, PPIA, DNMT3B) is activated, and the expression of exogenous transgenes is suppressed (Bayart and Cohen-Haguenauer, 2013; Karami et al., 2023).

Quantitative real-time PCR (qRT-PCR) is used to assess the gene expression levels: residual undifferentiated cells can be identified by the expression level of endogenous pluripotency-associated genes (Tano et al., 2014). In addition to the analysis of pluripotency-associated genes, qRT-PCR can be used to assess insertions and deletions, gene number variations, and single nucleotide polymorphisms (Zhong et al., 2022). For the integrative reprogramming strategies, qRT-PCR can be used to test the clearance of integrating vectors to avoid insertional mutagenesis (Rehakova et al., 2020). In addition to qRT-PCR, high-throughput expression assessment methods such as RNA-seq, scRNA-seq, and others are also used to assess the pluripotency-associated gene expression levels. However, due to the complexity of sample preparation and data processing, as well as the high costs, transcriptome analysis methods are less commonly used to assess the gene expression levels of iPSCs.

### 7.6 Methylation status assessment

Active DNA methylation remodeling occurs as a result of cell reprogramming (Cerneckis et al., 2024). In particular, the methylation status of cytosine-guanine dinucleotides (CpG) in the promoter regions of pluripotency-associated genes changes: in parental cells these are highly methylated, while in iPSCs the promoter regions are active and, hence, unmethylated (Takahashi et al., 2007). To assess the methylation level of CpG islands, bisulfite conversion approaches, restriction enzyme-based approaches (MSRE-PCR & MSRE-Southern Blot, COBRA), and affinity enrichment-based approaches (Methylation DNA immunoprecipitation assay, MeDIP, or MAP) can be used (Pajares et al., 2021). The most common method for assessing methylation status is bisulfite conversion, which is based on the chemical modification of unmethylated cytosine to uracil using bisulfite. Thus, the DNA sequence is modified depending on its methylation pattern. Bisulfite conversion-based approaches include a variety of methods, including methylation-specific PCR, bisulfite sequencing, droplet digital PCR, bisulfite pyrosequencing, and methylation-sensitive high-resolution technology (MS-HRM) (Pajares et al., 2021).

### 7.7 Analysis of iPSC differentiation

One of the critical characteristics of iPSCs is their ability to differentiate into the various cell types of the three germ layers. There are different approaches to assessing the level of pluripotency of iPSCs. The simplest method is spontaneous differentiation, when iPSCs are cultured as a suspension in the absence of FGF, resulting in the formation of dense structures consisting of multiple cell types called embryoid bodies (EBs), which then form the three germ layers, as assessed by immunofluorescent analysis (Rehakova et al., 2020).

Another option for assessing pluripotency is directed differentiation, during which iPSCs are exposed to differentiation media specific to each germ layer and then analyzed by immunofluorescence. Directed differentiation is a faster method of analysis and takes only a few days. A more time-consuming but traditional method of analyzing the pluripotent state is the teratoma formation test (Nelakanti et al., 2015), which involves the injection of undifferentiated iPSCs into the immunocompromised mice. After a few weeks, iPSCs form tumors that are histologically analyzed for tissue from all three germ layers. To date, this method of analysis is not considered appropriate, since it requires a lot of time for tumor growth, additional costs for animal work, and does not meet the ethical principles of using animals in cases where alternative methods exist.

Characterization of iPSCs and standardization of parameters will ensure the clinical use of these cells in cell therapy: the creation of a biobank of clinical-quality iPSC lines that comply with current good manufacturing practices (cGMP) seems especially relevant. Compared to the use of autologous iPSCs, biobanks use iPSCs from the HLA-compatible donors, which could be compatible with most recipients. The use of such biobanks is necessary in critical situations when it is not possible to carry out lengthy procedures associated with obtaining autologous iPSCs. For example, iPSCs from biobanks as an off-the-shelf product can be used for emergency care in the aftermath of myocardial infarction or spinal cord injury (Doss and Sachinidis, 2019). According to a recent review (Mah et al., 2023), the largest iPSC biobanks in the world are CIRM (California Institute for Regenerative Medicine, 2025), EbiSC (European Bank for induced pluripotent Stem Cells, 2025), RIKEN BRC (RIKEN BioResource Research Center, 2025), Sampled (Sampled, 2025), and WiCell (WiCell Research Institute, 2025); they contain collections of thousands of different iPSC variants.

## 8 Applications of iPSCs

**FIGURE 3:**
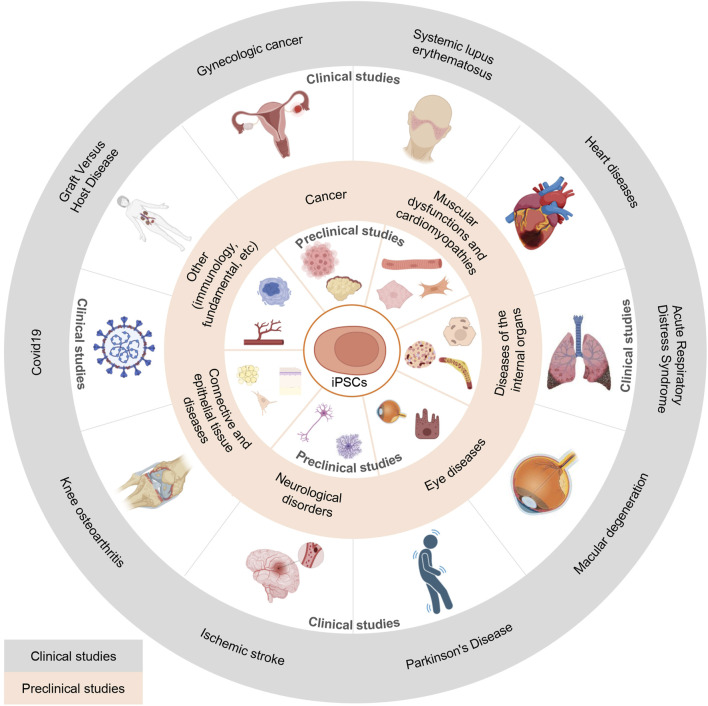
iPSC-based pre-clinical and clinical studies by therapeutic area.

Cells derived from iPSCs are widely used to model the development of various human diseases, conduct high-throughput drug screening, and develop autologous and allogeneic cell therapy options (Figure 3).

### 8.1 Regenerative cell therapy based on iPSCs

iPSC technology can be used to obtain hard-to-reach cell types and restore healthy tissue physiology after transplantation. iPSC-based cell therapies can be divided into two categories: autologous and allogeneic (Cerneckis et al., 2024). In the case of autologous cell therapy, iPSCs are obtained from the same patient who will undergo cell transplantation (Madrid et al., 2021; Schweitzer et al., 2021; 2020), which significantly reduces the risk of immune rejection of the graft by the recipient (graft-versus-host disease, GVHD). In the case of allogeneic cell therapy, iPSCs obtained from a universal donor are used for transplantation, which allows to significantly reduce the time and costs of producing individual iPSCs (Crow, 2019; Depil et al., 2020). Today, it is allogeneic cell therapy that has great potential for optimizing the production process.

### 8.2 Modeling diseases based on iPSCs

Traditionally, human diseases and their pathological mechanisms are studied using well-established *in vivo* animal models. However, there are significant interspecies differences between disease models (Liu Z. et al., 2018; Shi et al., 2017), which prevents an adequate assessment of the pathophysiology of the disease. This necessitates the development of human-specific disease models in addition to existing animal models (Wiegand and Banerjee, 2019). The main goal of iPSC-based disease modeling is to obtain cells with the required disease genotype and phenotype. There are two main approaches: isolating primary cells from donors with the corresponding disease and their subsequent reprogramming, or isolating and editing healthy cells using gene editing methods (e.g., CRISPR-Cas9) to obtain iPSCs with the disease genotype (Vuppalapu, 2023).

To date, a large number of iPSC-based disease models have been developed. It became possible to model neurological and psychiatric diseases (schizophrenia, autism spectrum disorders, Down syndrome, bipolar disorder, etc.), neurodegenerative diseases (Alzheimer’s disease, Parkinson’s disease, amyotrophic lateral sclerosis), (Cerneckis et al., 2024), spinal cord injuries (Gazdic et al., 2018; Kempermann et al., 2018; Takahashi J., 2018), and traumatic brain injuries, stroke (Cox, 2018; Liu G. et al., 2020; Niimi and Levison, 2018; Weston and Sun, 2018).

### 8.3 iPSC-based drug discovery platforms

Development of iPSC-based cell models has been widely used for high-throughput drug screening, including both phenotypic and targeted screening (Fatehullah et al., 2016; Shi Y. et al., 2017; Wiegand and Banerjee, 2019).

iPSC-derived cells can be used as a preclinical platform for testing drug efficacy and toxicity, as well as for identifying human-specific molecular mechanisms of drug action (Cerneckis et al., 2024).

When necessary, hard-to-reach cells can be obtained by reprogramming the cells of a patient with a specific disease. The effectiveness of the therapeutic agent is then assessed on the cells containing corresponding mutations that contribute to the pathological phenotype.

### 8.4 Reconstruction of organs

Efforts in recent years have been focused on the creation of various “organoid” (i.e., organ-like) models that are capable of reproducing *in vivo* the conditions of a tissue-specific environment (Brafman, 2013; Gattazzo et al., 2014). There are reports of successful creation of stomach and gastric organoids from mouse (Noguchi et al., 2015) and human ESCs and iPSCs (McCracken et al., 2014), liver organoids from hepatocytes obtained from iPSCs (Takebe et al., 2013), lung organoids from human ESCs (Dye et al., 2015; Miller et al., 2019), pancreatic islet organoids from pancreatic progenitor cells obtained from human PSCs (Candiello et al., 2018), retinal organoids (Eiraku et al., 2011; Kuwahara et al., 2017), and inner ear organoids (Koehler et al., 2017; 2013) from mouse ESCs and even cerebral organoids, which represent several brain regions with functional neurons (Lancaster et al., 2013). The ability to reproduce the structure of endogenous organs to study disease pathology in a spatio-temporal context and model the response to drugs at the organ level, rather than individual cells, determines the current popularity of organoids (Wiegand and Banerjee, 2019).

### 8.5 iPSC applications by therapeutic area

#### 8.5.1 Ocular diseases

In the field of regenerative therapy for ocular diseases, the five approved therapies are Holoclar®, Nepic®, Ocural®, Sakracy®, and Vyznova®. But eyes are a complex system; therefore, there are various stem cell-based strategies for treating ocular disorders. The main goals of ocular regenerative therapy are therapeutic effectiveness and the long-established transplantation of cells. Preclinical trials of iPSC-derived retinal pigment epithelial (RPE) transplantation have already started (Ishida et al., 2021; Rajendran Nair et al., 2021). Moreover, the cases of iPSC-derived RPE cell transplantation contributed to vision preservation. Other recent innovations in ocular regenerative medicine include the development of trabecular meshwork from mouse iPSCs (Sui et al., 2021; Wang X. et al., 2022) for glaucoma treatment. The development of new technologies related to regenerative medicine is active and consists of AMD and other ocular diseases (Supplementary Table S2); therefore, various graft forms are used depending on the disease and transplantation method, taking the various advantages of iPSCs.

#### 8.5.2 Nervous system disorders

Neurodegenerative disorders are among the most pressing medical issues of our time. The global burden of Alzheimer’s disease (AD) and Parkinson’s disease (PD) increases every year, and these and similar disorders (such as spinal cord injury (SCI) and Duchenne muscular dystrophy (DMD)) need effective therapy in clinical practice. In this context, iPSCs are a promising experimental approach that may open a window to the development of effective therapies. Animal studies have proven the possibility of transplantation of iPSC-derived nervous cells into lesions against the background of the AD (Pomeshchik et al., 2023). The PD manifestation appears through dopaminergic neuronal loss and the presence of Lewy bodies, and usage of iPSCs is popular in PD therapy development (Brot et al., 2022; Doi et al., 2020). Up until recently, SCI was believed to be an incurable condition, but transplantation of neural precursor cells gained remarkable attention as a reasonable therapeutic intervention to replace the damaged central nervous system cells and promote functional recovery in animals (Kawai et al., 2021; Kim et al., 2024; Liu A. et al., 2020; Shibata et al., 2023; Zheng et al., 2022).

#### 8.5.3 Cancer

Considering the fact that iPSC lines can be tumorigenic after grafting, it is important to take measures to lower this risk (Nagoshi et al., 2019). Despite this, iPSCs found their way into cancer therapy as well. Cancer immunotherapy based on harnessing the power of the immune system to selectively target and eliminate cancer cells can also rely on iPSCs, which can offer an unlimited source of immune cells for manipulation and expansion. Some promising strategies include the generation of iPSC-derived natural killer (NK) cells and macrophages (Cichocki et al., 2020; Li S. et al., 2024). *In vivo* studies have already demonstrated effective tumor regression. Therapeutic cancer vaccines, which induce specific effector cells to eliminate cancer cells, have elicited renewed interest due to the development of the iPSC platform (Gąbka-Buszek et al., 2020; Huang et al., 2024; Hundt et al., 2021; Ouyang et al., 2021). The iPSC-based cancer vaccines not only prevented tumor growth and metastasis but also induced a cytotoxic antitumor response.

#### 8.5.4 Diseases affecting internal organs

iPSC-based therapy has recently emerged as a promising approach to treat disorders affecting internal organs (Gaykema et al., 2024; Hogrebe et al., 2020; Hu X. et al., 2024; Lau et al., 2020; Li H. et al., 2024; Nagata et al., 2020). For example, transplanted human endocrinologically active pancreatic islet cells and stem cell-derived pancreatic β (SC-β) cells in diabetic mice (Hogrebe et al., 2020; Hu et X. al., 2024) secreted insulin and controlled glycemia. Similarly, human iPSC-derived cells can be successfully used for the therapy of kidney diseases. iPSC-derived kidney organoids and podocytes (iPSC-PODs) survived in recipient mice, but it was necessary to prevent rejection following transplantation (Gaykema et al., 2024; Lau et al., 2020). Hepatobiliary organoids (HBOs) and iPSC-hepatocytes (REPROCELL) were used for the treatment of liver diseases in cynomolgus monkeys and mouse models (Li H. et al., 2024; Nagata et al., 2020). These examples indicate that the HBO transplantation improved hepatoprotection effects; furthermore, the transplantation of iPSC-derived microfibers led to the detectable functional activity of iPSC-derived hepatocytes. As a result, various clinical trials using iPSC-derived cellular products for the treatment of human diseases have been initiated and are presented in Supplementary Table S3.

#### 8.5.5 Muscle dysfunctions and cardiomyopathies

The muscles are the major organ system in human bodies. Their function can be affected by either genetic diseases or various injuries. Muscle regeneration is a well-adjusted process; however, it can be insufficient or become exhausted by the ongoing fiber damage that, for example, occurs in muscular dystrophy. iPSC-based therapy provides a source of myogenic cells that repopulate the lost muscle fibers. Duchenne Muscular Dystrophy is a recessive form of muscular disorder resulting from the dystrophin gene mutations in the X chromosome. In a study by Miura and colleagues (2022), muscle stem cells (MuSCs) were transplanted into the mouse diaphragm and successfully engrafted into the diaphragm. Another study (Guo et al., 2022) demonstrated the regulatory plasticity of iPCS-derived skeletal muscle myoblasts (iMyoblasts) for adult muscle maturation in response to signals in the host’s muscle. Diseases such as myocardial infarction and heart failure were also treated with the help of iPSC-derived cardiomyocytes (Guan X. et al., 2020; Lou et al., 2020; Yoshida S. et al., 2020). Such therapies led to an increase in the effectiveness of cardiac function in animal studies.

#### 8.5.6 Skin, cartilage, bones, joints

Usually bone defects result from severe fractures in elderly osteoporosis patients, trauma, tumor ablation, or congenital abnormalities, which creates a great need for an alternative to autologous grafts for such patients. iPSC-based regenerative therapy offers new therapeutic options for patients with bone defects. Recently, the efficiency of establishing iPSCs has been confirmed on mouse models (Agten et al., 2022; Satake et al., 2022). However, as iPSCs and their derivatives are not sufficient to solve this problem, the field of tissue engineering is increasingly focused on the combined use of biomaterials to build or regrow patient-specific tissues, such as bone (Abe et al., 2023; Kessler et al., 2024). Usage of 3D-printing methods can be a promising alternative for the treatment of narrowing and stenosis of the upper airway (Kim et al., 2020). 3D-printed trachea combined with various cell types was shown to regenerate into a functional airway on the New Zealand white rabbit model. Also, iPSCs can be directed towards the differentiation into epidermal keratinocytes, one of the cell types often affected in skin disorders. iPSC-derived keratinocytes can then be used for the generation of a stratified epidermis *in vivo* (iPSC-derived skin cell suspension liquid transplantation into mice) (Ebner-Peking et al., 2021).

#### 8.5.7 Other

There are many options for the application of iPSCs as both *in vitro* and *in vivo* models. For example, the prevalence of diseases associated with nerve cell damage makes it necessary to have relevant models of such diseases (Hasselmann et al., 2019). Cancerous tumors are often heterogeneous, making screening of therapeutic molecules ineffective, as was demonstrated in one of the published studies on AML (Kotini et al., 2023). Disorders of the cardiovascular system are among the most common diseases; fortunately, cell-based models that allow effective drug screening have already been described (Li et al., 2021). The use of animal models for human immunodeficiency virus type-1 (HIV-1) diagnosis (Min et al., 2023) or for xenotransplantation (Song et al., 2021) is also becoming possible as a result of iPSCs technology. One of the common problems with transplants is the subsequent rejection of the graft (a condition known as GVHD), and the published study on the engraftment of the kidney organoid sheds light on the interaction of the graft and human immune cells (Shankar et al., 2024). Rejection of cell therapies by the host’s immune system remains a major problem for regenerative medicine, the solution to which, in addition to the traditional immunosuppressants, can be found in engineering hypoimmune cells or possibly the infusion of Treg cells (regulatory T lymphocytes) that dampen the immune response. For instance, a new class of agonistic immune checkpoint engagers that protect human leukocyte antigen (HLA)-depleted iPSCs-derived endothelial cells (iECs) from innate immune cells was presented recently (Gravina et al., 2023). In the future, research in this area will greatly advance cell therapeutics. While there are still many opportunities for the application of iPSC technology for those diseases that are yet to benefit from it, there is already data about attempts to cure such diseases as premature ovarian insufficiency (POI) and thrombocytopenia (Elias et al., 2023; Sugimoto et al., 2022).

### 8.6 Clinical studies

iPSCs have a great potential as a therapeutic approach to regenerate or replace functionally impaired tissues. Recent years saw a gradual increase in the number of scientific studies on this topic. However, no PSC-based therapy has found its way into routine clinical use so far. The search of the clinical trial (CT) database (ClinicalTrials.gov, 2025) identified eighty-nine trials using the keywords “induced pluripotent stem cell”. Forty-seven CTs (52.8%) are observational and nontherapeutic, whereas thirty-nine CTs (43.8%) are interventional and therapeutic. As not all of the therapeutic studies rely on iPSCs, nineteen CTs were eliminated. Of the remaining twenty studies presented in Supplementary Table S3, three were terminated, and one had an undisclosed status. Half of the twenty interventional trials (40%) use allogeneic iPSCs, while autologous iPSCs are used in six CTs (30%). Nine studies (45%) are in Phase I, and three studies (15%) are classified as Phase I/II CTs. No phase-related information is available for the remaining three studies.

Geographically, the studies are being conducted in six countries: Australia, China, France, Japan, India, and the United States. As much as 40% of trials (eight out of twenty) are conducted in the USA, and 35% (seven out of twenty) are conducted in China.

The most studied conditions are cardiovascular diseases (4 trials) and Parkinson’s disease (4 trials). The rest of the studies focus on macular degeneration, cancer, GvHD, and other conditions. Eight CTs study the effect of transplantation of either iPSC-derived MSCs or iPSC-derived dopaminergic neurons. The researchers attempted to treat macular degeneration with iPSC-derived RPE cells in three trials. Human iPSC-derived cardiomyocytes are used in two studies for the treatment of cardiovascular diseases, while iPSCs of the cardiac lineage and iPSC-derived cardiomyocyte spheroids are each used in one trial. iPSC-derived natural killer cells (iNK) are used against tumors and gynecological cancer in two CTs. The enrollment in these CTs ranges from 3 to 60 participants, with the average number of 24 (SE = 3.9) (Supplementary Table S3).

### 8.7 Biobanks of iPSC cells around the world

With the development of the iPSC platform, there are now several biobanks that offer their services.

The Prader-Willi syndrome (PWS) iPSC Biobank is a biobank that has three iPSC lines from people with the most common genetic cause of life-threatening childhood obesity, PWS. These iPSC lines are open for academia and industry worldwide and are verified by the set of validation assays. PWS large deletion line 1.7 (PWS1.7) was reprogrammed into iPSCs using RV vectors encoding Oct4, Sox2, Klf4, c-Myc, and Lin28 (Chamberlain et al., 2010), PWS small atypical deletion line 2.9 (PWS2.9) was reprogrammed into iPSCs using a polycistronic STEMCCA LV vector encoding Oct4, Klf4, Sox2, and c-Myc (Martins-Taylor et al., 2014), PWS maternal uniparental disomy line 1.2 (PWSUPD1.2) was reprogrammed into iPSCs using RV vector as well (Sommer et al., 2009).

The European Bank of induced pluripotent Stem Cells (EBiSC) has a large collection of well-characterized human iPSC lines from a range of genetic backgrounds obtained by various reprogramming methods. There are many types of iPCS lines with *APOE* gene mutations (a critical risk for developing Alzheimer’s disease) reprogrammed via episomal vectors and Sendai virus delivering the transcription factors (Schmid et al., 2021; 2019). In addition, there is a panel of robust and well-characterized iPSC lines from healthy donors (reprogrammed via episomal, RV, and SeV vectors).

The iPSC biobank, which is a part of the Amsterdam UMC biobank, can generate transgene-free iPSCs by using SeV vectors, which provides a safer non-integrating alternative to the traditional reprogramming methods. After reprogramming, the iPSC lines can be stored in this biobank.

REPROCELL iPSC biobank (mainly located in the USA) has several iPSC lines, which were reprogrammed using StemRNA™ 3rd Gen Reprogramming Technology and are subject to a thorough quality control process.

The iPSC collection from allogeneic healthy donors could be a good alternative to the patient-specific iPSCs. As described previously (Kuebler et al., 2023), seven donors were selected covering 21.37% of the Spanish population haplotypes. Purified CD34^+^ cells were reprogrammed by transduction with SeV vectors.

Several banks of HLA-homozygous iPSCs (haplobanks) have already been established worldwide or are underway to provide clinical-grade starting material for cell therapies covering the most frequent HLA haplotypes for certain population groups.

The Center for iPS Cell Research and Application (CiRA) at Kyoto University was selected as the core center for the iPSC Stock Project, the goal of which is to manufacture and release an HLA homozygous iPSC stock that can cover almost the entire Japanese population. iPSCs in this bank were obtained by the electroporation of episomal plasmid vectors that introduce the reprogramming genes (Hanatani and Takasu, 2021).

The CHA Stem Cell Institute, at CHA University in South Korea, published a haplobank with Korean haplotypes (reprogramming into iPSCs via episomal plasmids) (Lee S. et al., 2018).

## 9 Discussion

The ability to reprogram already differentiated cells into iPSCs paved the way towards the development of *in vitro* models of various human pathologies, high-throughput drug discovery, and autologous and allogeneic cell and regenerative therapy. This would be impossible to achieve without the accumulation of significant knowledge in both iPSC research and many areas of molecular biology made in the last 20 years. However, the production of iPSC-based therapies is hampered by the low reprogramming efficiency, the complexity of characterization, and the concerns about the safety profile of this approach.

Various insightful iPSC-based studies demonstrated that the reprogramming success depends on several key factors: the source of cells used for induction of pluripotency, the age of donors, the combination and ratio of transcription factors, delivery approaches, and additional factors, such as miRNAs and small chemical compounds used to either enhance the effect or substitute certain TFs.

Overall, the process of cell reprogramming is associated with remodeling of chromatin structure and changes in the epigenome, as well as changes in cell metabolism, signaling, intracellular transport, proteostasis, and many other cell biology processes. The association of c-Myc with histone acetyltransferase complexes induces global histone acetylation and promotes enhanced reprogramming due to the presence of the vast number of binding sites. This step is followed by the binding of key transcription factors, Oct4 and Sox2, which inhibit the expression of genes associated with ESC differentiation to their specific target sites (Gillis et al., 2011; Karami et al., 2023; Takahashi K. and Yamanaka, 2006). The reprogramming efficiency and the quality of iPSC colonies depend on the expression levels and the ratio of TFs (Fus-Kujawa et al., 2021; Gillis et al., 2011; Matsuoka et al., 2012; Yamaguchi et al., 2011). Klf4 both inhibits the expression of a large number of genes specific to intermediately reprogrammed cells and induces the expression of genes associated with pluripotency (Kulcenty et al., 2015). Importantly, the expression of exogenous reprogramming factors, while crucial during the first stages of reprogramming, should not be maintained continuously.

In addition to c-Myc, Oct4, Sox2, and Klf4, transcription factors Nanog and Lin28 were described as important factors contributing to the induction and maintenance of pluripotency (Boyer et al., 2005; Liu G. et al., 2020; Loh et al., 2006). For example, Lin28 was proposed to act as a c-Myc substitute as it accelerates cell proliferation and affects the early phase of iPSC generation (Golipour et al., 2012). Different combinations and ratios of these factors yield various degrees of reprogramming efficiency (Bailly et al., 2022; Lapasset et al., 2011; Liao et al., 2008). And even though Nanog and Lin28 were previously described as effective analogs of Klf4 and c-Myc, it was their addition to the traditional Yamanaka cocktail that resulted in a 10-fold increase in reprogramming efficiency of fibroblasts compared to the combination of factors that lacked Klf4 and c-Myc (OSNL) and even led to the successful reprogramming of fibroblasts from old donors. On the other hand, a three-factor cocktail (Oct4, Sox2, and Esrrb) led to the greater efficiency of reprogramming of mouse embryonic fibroblasts than the Yamanaka cocktail (Feng et al., 2009b). Interestingly but not unexpectedly, cells that already have endogenous expression of some key TFs can be reprogrammed with only Oct4 (Kim et al., 2009a; 2009b), Sox2 (Utikal et al., 2009), or Oct4 and Sox2 (Giorgetti et al., 2009; Meng et al., 2012).

Another key factor affecting the reprogramming efficiency is the delivery of the TFs into the cells, which can be achieved by viral or non-viral means and includes integrative and non-integrative transient methods. The advantage of viral (e.g., LV and AAV)-based methods is the high efficiency of gene delivery to various types of somatic cells as compared to transfection and higher cell viability as compared to electroporation, for example,. Stable gene expression at high levels during the initial stages, necessary for successful reprogramming, can be achieved by using integrating viruses, such as LV. However, the constant high expression of exogenous pluripotency factors is only a temporary requirement. This drawback can be resolved by the application of the Tet-inducible system, which ensures the repression of pluripotency factors in the iPSC-like colony. SeV-based vectors that do not possess the ability to integrate into the host cell genome and express genes at high levels during the initial stages of reprogramming became popular not only for this important reason but also due to their cellular tropism, ease of use, and high efficiency. A disadvantage of this method is that SeV vector elimination from the cell takes about ten passages. However, the number of vector copies of the temperature-sensitive SeV version can be drastically decreased by changing the temperature (Nishishita et al., 2012; Ono et al., 2012).

The reprogramming cassette can be removed in the case of two PB- and SB-based transposon systems; however, the low efficiency of DNA transfection of some primary cell lines and the potential risk of uncontrolled off-target transposition can be limiting factors to using this approach (Bayart and Cohen-Haguenauer, 2013; Wang et al., 2008).

Episomal vectors based on the oriP/Epstein-Barr nuclear antigen-1 can replicate as extrachromosomal elements, are maintained as stable episomes under the control of a selective inducer, and can be eliminated upon its removal (Yates et al., 1984; 1985). These vectors can be successfully used to reprogram a variety of cell types (skin fibroblasts, blood cells, MSCs, etc.) and are relatively inexpensive; however, the efficiency of cell reprogramming is quite low.

The advantage of using mRNA compared to plasmid DNA is the immediate translation in the cytoplasm, safety compared to the integrative viral vectors such as LV) as RNA does not integrate into the host cell genome, and efficiency compared to other non-viral, non-integrating delivery systems (Bailly et al., 2022). However, the rapid degradation of mRNA in the cytoplasm significantly decreases the expression levels of the delivered pluripotency factors, resulting in the low efficiency of reprogramming (Cherkashova et al., 2020). Therefore, successful and efficient mRNA-based reprogramming protocols rely on several rounds of transfections (Yakubov et al., 2010). It is also important to note that synthetic mRNAs are capable of activating the innate immune system, which suppresses protein translation and triggers a cascade of cytotoxic reactions (Warren and Lin 2019). The disadvantages of the mRNA approach can be solved by the optimization of the 5′ and 3′UTRs, the polyA tail, a synthetic cap analog, and the incorporation of modified uridine analogs (Bayart and Cohen-Haguenauer, 2013).

Reprogramming of somatic cells is possible not only with the help of exogenous transcription factors but also by the addition of small molecule inducers of pluripotency. AZA (Mikkelsen et al., 2008), VPA (Huangfu et al., 2008a), sodium butyrate (Mali et al., 2010), parnate (Li H. et al., 2009) and some others can compensate for or enhance the action of individual pluripotency factors. Overall, it seems possible to speculate that the combination of the minimal number of transcription factors with effective molecules inducers of pluripotency should yield the greatest reprogramming efficiency.

The traditional cultivating protocol involves culturing iPSCs on a monolayer of mitotically inactivated feeder cells that secrete important growth factors, extracellular matrix components, and cytokines into the medium (Dakhore et al., 2018; Sams and Powers, 2013; Yao et al., 2006). This method, however, is labor-intensive, difficult to scale up, may present safety issues due to the potential presence of pathogens and *mycoplasma* contamination, and may complicate the characterization of the iPSCs. Therefore, the development of protocols for iPSC cultivation in the absence of a feeder layer (such as those using laminin coating) ensures the reliability, reproducibility, sustainability, efficiency, and safety of the process, facilitates scaling up, and enables high-throughput screening.

In conclusion, even though it took over 20 years for the complex field of iPSC research to develop, the accumulated valuable knowledge paved the way for the various iPSC applications. This technology can now be used both for regenerative therapy purposes and for *in vitro* disease modeling to study human pathologies and test new therapies. The discussed difficulties in obtaining iPSCs do not overshadow or prevent their production; on the contrary, finding the solutions to overcome these obstacles brought the biomedical research towards a deeper understanding of molecular medicine, successful treatment of various human diseases, and saving patient lives.

## Glossary

iPSCs: induced pluripotent stem cells
hiPSC: human induced pluripotent stem cells
SCNT: somatic cell nuclear transfer
OSKM: Oct4, Sox2, Klf4 and c-Myc, “Yamanaka factors” or “Yamanaka cocktail”
OSNL: Oct4, Sox2, Nanog and Lin28
OSKML: Oct4, Sox2, Klf4, c-Myc and Lin28
OSKMNL: Oct4, Sox2, Klf4, c-Myc, Nanog and Lin28
OSM: Oct4, Sox2 and c-Myc
OK: Oct4 and Klf4
OS: Oct4 and Sox2
SKM: Sox2, Klf4 and c-Myc
MEFs: mouse embryonic fibroblasts
HLA: human leukocyte antigen
AZA: 5-azacytidine
VPA: valproic acid
LV: lentiviruses
Ad: adenoviruses
AAV: adeno-associated viruses
HSV: herpes viruses
GLAd: “gutless” adenoviruses
HF-GLAd: helper virus-free gutless adenovirus
SeV: Sendai virus
SeVdp: Sendai virus replication-deficient vector
ITRs: inverted terminal repeats
SB: “Sleeping Beauty” transposons
PB: Piggybac transposons
oriP/EBNA1: oriP/Epstein-Barr nuclear antigen-1
mRNA: messenger RNA
saRNA: self-replicating RNA
GMP: good manufacturing practice
cGMP: current good manufacturing practices
ESCs: embryonic stem cells
VLPs: virus-like particles
MET: mesothelial-to-epithelial transition
EMT: pro-epithelial-to-mesenchymal transition
GSK3: glycogen kinase 3
MEK(MAPK)/ERK: mitogen-activated protein kinase/extracellular signal-regulated kinase 1/2
H3K9me3: trimethylation at lysine 9 of histone H3
HDAC: histone deacetylases
NaB: sodium butyrate
FGFs: fibroblast growth factors
bFGF: basic fibroblast growth factor
ROCK: Rho-associated protein kinase
CiPSCs: chemically induced pluripotent stem cells
DZNep: 3-deazaneplanocin A
BrdU: bromodeoxyuridine
F6P: fructose-6-phosphate
2-DG: 2-deoxy-D-glucose
HIF1α: hypoxia-inducible factor 1-alpha
HSCs: hematopoietic stem cells
MNCs: blood mononuclear cells
PBMCs: peripheral blood mononuclear cell
MSCs: mesenchymal stem cells
NPCs: neural progenitor cells
RCA: replication-competent adenovirus
FCB: fluorescence cell barcoding
FACS: fluorescence-activated cell sorting
MACS: magnetic activation cell sorting
AP: with alkaline phosphatase
aCGH: array comparative genomic hybridization
SNP: single nucleotide polymorphism
STR: short tandem repeat
NGS: next-generation sequencing
WGS: whole-genome sequencing
WES: whole-exome sequencing
qRT-PCR: quantitative real-time PCR
RNA-seq: RNA sequencing
scRNA-seq: single cell RNA sequencing
CpG: cytosine-guanine dinucleotides
MS-HRM: methylation-sensitive high-resolution technology
EBs: embryoid bodies
CRISPR: clustered regularly interspaced short palindromic repeats
RPE: retinal pigment epithelium
AMD: age-related macular degeneration
RCS: Royal College of Surgeons
LHON: leber hereditary optic neuropathy
OCT: optical coherence tomography
TM: trabecular meshwork
DAPs: dopaminergic progenitors
PD: Parkinson’s disease
6-OHDA: 6-hydroxydopamine
SNpc: substantia nigra pars compacta
SCI: spinal cord injury
NOD-SCID mice: nonobese diabetic severe combined immune deficient mice
NSCs: neural stem cells
HPCs: hematopoietic progenitor cells
ALSP: adult-onset leukoencephalopathy with axonal spheroids and pigmented glia
AD: Alzheimer’s disease
HD: Huntington’s disease
MNP: motor neuron progenitor cells
MN: motor neuron
SD: Sprague-Dawley
UiPSC: urine derived iPSCs
PTSD: post-traumatic stress disorder
NK: natural killer
PDAC: pancreatic ductal adenocarcinoma
CRC: colorectal cancer
TNBC: triple-negative breast cancer
NA-iPSCs: neoantigen-augmented iPSCs
miPSCs: murine induced pluripotent stem cells
TLR: Toll-like receptor
SC-β cells: stem cell-derived pancreatic β cells
HBOs: hepatobiliary organoids
DMD: Duchenne muscular dystrophy
MuSCs: muscle stem cells
CMs: cardiomyocytes
GelMA: gelatin methacryloyl hydrogel
ECFCs: endothelial colony forming cells
FBs: skin fibroblasts
KCs: keratinocytes
cyiPSCs: cynomolgus monkey iPSCs
BMMCs: bown marrow mononuclear cells
AML: acute myeloid leukemia
FAD: familial Alzheimer’s disease
EC: endothelial cells
HIV-1: human immunodeficiency virus type-1
POI: premature ovarian insufficiency
PLT: platelet
CT: clinical trial
aGvHD: acute graft *versus* host disease
KOA: knee osteoarthritis
GA: geographic atrophy
d-AMD: dry age-related macular degeneration
CIRM: California Institute for Regenerative Medicine
EBiSC: European Bank for induced pluripotent Stem Cells
RIKEN BRC: RIKEN BioResource Research Center
PWS: Prader-Willi syndrome
Amsterdam UMC biobank: Amsterdam University Medical Center biobank
CiRA: The Center for iPS Cell Research and Application.
