# Supplementary material for: A comprehensive analysis of induced pluripotent stem cell (iPSC) production and applications
Source: Front Cell Dev Biol. 2025 May 8;13:1593207. doi: 10.3389/fcell.2025.1593207 (PMC12095295; doi:10.3389/fcell.2025.1593207)
Supplement: Supplementary file 1 [file Table1.docx]

**Table S1. Efficiency of reprogramming methods depending on the cell types**

| Reprogramming methods | | Expected levels of reprogramming efficiency | | | | | | | Possible Improvements |
| --- | --- | --- | --- | --- | --- | --- | --- | --- | --- |
|  |  | Skin cells | Blood cells | | Stem cells | | Other cell types | |  |
| **Viral transduction** | Retroviruses | Fibroblasts:  **< 0.01%** _(Aasen et al., 2008)_ – **~1%** _(Huangfu et al., 2008)_  Keratinocytes: **0.04%** _(Aasen et al., 2008)_ – **1.89%** _(Giorgetti et al., 2009)_ | PBMC (CD34+):  [ND] _(Loh et al., 2009)_    PBMC: [ND] _(Kunisato et al., 2011)_    Menstrual blood cells: [ND] _(Nishino et al., 2018)_ | | Neural stem cells: **0.004% – 0.006%** _(Kim J. B. et al., 2009a)_    Adipose Stem Cells: **0.008%** – **1.5%** _(Sugii et al., 2011)_    Umbilical cord matrix mesenchymal cells: **0.4%** _(Cai et al., 2010)_    Amniotic membrane mesenchymal cells: **0.1%** _(Cai et al., 2010)_ | | Urine cells:  **0.01** – **4%** _(Zhou T. et al., 2011)_  Placental artery endothelium, amnion, endometrium: [ND] _(Nishino et al., 2011)_    Conjunctival epithelial cells:  **1.5%** _(Poon et al., 2015)_    Conjunctival-derived stromal cells:  **0.1%** _(Poon et al., 2015)_ | | **Cre/loxP** or **FLP-FRT** systems contribute towards transgene removal.    **Tetracycline/doxycycline**-inducible vector systems allow the control of transgene expression. |
|  | Lentiviruses | Keratinocytes:  **0.002%** _(W. Li et al., 2009)_ – **2.8%** _(Linta et al., 2012)_    Melanocytes: **0.05%** _(Utikal et al., 2009)_ | | PBMC (CD34+): **0.002%** _(Loh et al., 2010)_    PBMC:  **0.0002%** _(Staerk et al., 2010)_ – **0.01%** _(Ohmine et al., 2011)_    Cord Blood Cells: **0.0001%** – **0.03%** _(Haase et al., 2009)_ | | Adipose Stem Cells:  **0.2%** _(Sun et al., 2009)_ | | Urine cells:  **0.002 –** **0.5%** _(Guan X. et al., 2014)_ |  |
|  | Sendai viruses | Keratinocytes: **0.002** – **0.01%** _(Nakayama et al., 2018)_    Dermal fibroblasts, fibroblasts BJ:  **1%** _(Fusaki et al., 2009)_    Neonatal fibroblasts:  **0.01** **– 0.1%** _(Lieu et al., 2013)_    Fibroblasts**: ~ 0.1%** _(Schlaeger et al., 2015)_ | | Terminally differentiated circulating T cells:  **0.1%** _(Seki et al., 2010)_    PBMCs (CD34+):  **5.58%** _(Okumura et al., 2019)_    PBMCs: **0.0015%**  _(Kim Y. et al., 2016)_ **– 0.5%** _(Sharma A. et al., 2018)_    Cord blood (CD34+) cells:  [ND] _(Takayama et al., 2010)_    Menstrual blood cells: [ND] _(Nishino et al., 2018)_ | | Amniotic fluid mesenchymal stromal cells: **0.01 – 0.05%** _(Jiang G. et al., 2014)_    Dental mesenchymal stem cells: **0.2%** _(Tan et al., 2018)_ | | Urine cells:  **0.001 – 0.1%** _(Liu Y. et al., 2017)_    Skeletal Myoblasts: **0.75%** _(Trokovic et al., 2014)_    Nasal epithelial cells:  **0.08 – 0.10%** _(Ono et al., 2012)_    Umbilical vein endothelial cells: [ND] _(Nishio and Saeki, 2014)_ | Replication-defective Sendai virus **(SeVdp)** leads to persistent transgene expression (Nishimura et al., 2011).    Temperature sensitive Sendai virus vector **(SeV TS7)** was developed to improve vector elimination (Nishishita et al., 2012; Ono et al., 2012). |
|  | Adenoviruses | Fibroblasts: **0.0002%** _(Zhou W. and Freed, 2009)_ | | – | | – | | – | Helper virus-free gutless adenovirus **HF-GLAd** has large transgene cargo capacity and high safety profile (no replication-competent adenovirus (RCA)) (Liu J. and Seol, 2020). |
| **Non-viral delivery** | Episomal vectors (plasmids, minicircles) | Keratinocytes:  **0.14%** _(Piao et al., 2014)_    Fibroblasts (plasmids): **0.00033%** _(Si-Tayeb et al., 2010)_    Fibroblasts (minicircles):  **0.005%** _(Diecke et al., 2015)_ | | PBMC (CD34+):  **0.0005%** _(Okita et al., 2013)_ **– 0.2%** _(Su et al., 2013)_    PBMC:  **0.00025%** _(Diecke et al., 2015)_ **– 0.2%** _(Wen et al., 2016)_    Menstrual blood cells: [ND] _(Nishino et al., 2018)_ | | Adipose derived stem cells (plasmid):  **0.006% – 0.01%** _(Qu et al., 2012)_    Adipose derived stem cells (minicircles):  **~ 0.005%** _(Narsinh et al., 2011)_ | | Urine cells:  **0.0001%** _(Xue et al., 2013)_ – **1.5%** _(Drozd et al., 2015)_    Dental pulp:  [ND] _(Okita et al., 2011)_ | **oriP/Epstein–Barr** nuclear antigen-1-based episomal vectors can be removed from cells in the absence of drug selection (Yu et al., 2009). |
|  | Synthetic RNAs | Foreskin fibroblasts: **0.012** _(Yoshioka et al., 2013)_ **– 4.4%** _(Warren et al., 2010)_    Embryonic fibroblasts: **1.4 % – > 2.5%** _(Warren et al., 2010)_    Fibroblasts: **~ 1%** _(Schlaeger et al., 2015)_ | | – | | – | | Urine cells: **0.008 – 0.17%** _(Bouma et al., 2020)_ | Modified **5’-caps** (m7GpppG, ARCA) and modified **nucleotide equivalent** (5mU, 5mC, ψ), 6mA, 1mψU, 5moU) could increase mRNA stability and lifespan and reduce immune response (Bailly et al., 2022).    **B18R** (inhibitor of interferon-mediated antiviral activity) could suppress immune response (Poleganov et al., 2015).    **Self-replicative RNA** can be used for single transfection. |
|  | Transposons | Keratinocytes: **~ 1%** _(Igawa et al., 2014)_    Fibroblasts: **~ 0.01%** _(Grabundzija et al., 2013)_ | | – | | – | | – | Translocation activity of **PiggyBac** is significantly higher than SleepingBeauty in mammalian cell lines (Wu S. C.-Y. et al., 2006) and does not leave a genetic trace in the host genome (Woltjen et al., 2011).    **Hyperactive transposase** (SB100X (Mátés et al., 2009) or hypPB (Doherty et al., 2012)) demonstrates higher transposition activity.    Switching the transposase source **from plasmid DNA to mRNA** could mitigate the toxicity of the delivery and decrease the risk of remobilization of the integrated transgene (Narayanavari and Izsvák, 2017). |
|  | Proteins | Fibroblasts: **0.001%** _(Kim D. et al., 2009)_ | | – | | – | | – | **Reversible permeabilization** (using streptolysin O or cationic bolamphiphiles) could improve protein transport through plasma membrane (Bailly et al., 2022). |

References:

Cai, J., Li, W., Su, H., Qin, D., Yang, J., Zhu, F., et al. (2010). Generation of Human Induced Pluripotent Stem Cells from Umbilical Cord Matrix and Amniotic Membrane Mesenchymal Cells. *J. Biol. Chem.* 285, 11227–11234. doi: 10.1074/jbc.M109.086389

Diecke, S., Lu, J., Lee, J., Termglinchan, V., Kooreman, N. G., Burridge, P. W., et al. (2015). Novel codon-optimized mini-intronic plasmid for efficient, inexpensive and xeno-free induction of pluripotency. *Sci. Rep.* 5, 8081. doi: 10.1038/srep08081

Doherty, J. E., Huye, L. E., Yusa, K., Zhou, L., Craig, N. L., and Wilson, M. H. (2012). Hyperactive *piggyBac* Gene Transfer in Human Cells and *In Vivo*. *Hum. Gene Ther.* 23, 311–320. doi: 10.1089/hum.2011.138

Drozd, A. M., Walczak, M. P., Piaskowski, S., Stoczynska-Fidelus, E., Rieske, P., and Grzela, D. P. (2015). Generation of human iPSCs from cells of fibroblastic and epithelial origin by means of the oriP/EBNA-1 episomal reprogramming system. *Stem Cell Res. Ther.* 6, 122. doi: 10.1186/s13287-015-0112-3

Fusaki, N., Ban, H., Nishiyama, A., Saeki, K., and Hasegawa, M. (2009). Efficient induction of transgene-free human pluripotent stem cells using a vector based on Sendai virus, an RNA virus that does not integrate into the host genome. *Proc. Jpn. Acad. Ser. B* 85, 348–362. doi: 10.2183/pjab.85.348

Grabundzija, I., Wang, J., Sebe, A., Erdei, Z., Kajdi, R., Devaraj, A., et al. (2013). Sleeping Beauty transposon-based system for cellular reprogramming and targeted gene insertion in induced pluripotent stem cells. *Nucleic Acids Res.* 41, 1829–1847. doi: 10.1093/nar/gks1305

Guan, X., Mack, D. L., Moreno, C. M., Strande, J. L., Mathieu, J., Shi, Y., et al. (2014). Dystrophin-deficient cardiomyocytes derived from human urine: New biologic reagents for drug discovery. *Stem Cell Res.* 12, 467–480. doi: 10.1016/j.scr.2013.12.004

Jiang, G., Di Bernardo, J., Maiden, M. M., Villa-Diaz, L. G., Mabrouk, O. S., Krebsbach, P. H., et al. (2014). Human Transgene-Free Amniotic-Fluid-Derived Induced Pluripotent Stem Cells for Autologous Cell Therapy. *Stem Cells Dev.* 23, 2613–2625. doi: 10.1089/scd.2014.0110

Igawa, K., Kokubu, C., Yusa, K., Horie, K., Yoshimura, Y., Yamauchi, K., et al. (2014). Removal of Reprogramming Transgenes Improves the Tissue Reconstitution Potential of Keratinocytes Generated From Human Induced Pluripotent Stem Cells. *Stem Cells Transl. Med.* 3, 992–1001. doi: 10.5966/sctm.2013-0179

Kim, Y., Rim, Y. A., Yi, H., Park, N., Park, S.-H., and Ju, J. H. (2016). The Generation of Human Induced Pluripotent Stem Cells from Blood Cells: An Efficient Protocol Using Serial Plating of Reprogrammed Cells by Centrifugation. *Stem Cells Int.* 2016, 1329459. doi: 10.1155/2016/1329459

Kunisato, A., Wakatsuki, M., Shinba, H., Ota, T., Ishida, I., and Nagao, K. (2011). Direct Generation of Induced Pluripotent Stem Cells from Human Nonmobilized Blood. *Stem Cells Dev.* 20, 159–168. doi: 10.1089/scd.2010.0063

Lieu, P. T., Fontes, A., Vemuri, M. C., and MacArthur, C. C. (2013). “Generation of Induced Pluripotent Stem Cells with CytoTune, a Non-Integrating Sendai Virus,” in *Pluripotent Stem Cells*, eds. U. Lakshmipathy and M. C. Vemuri (Totowa, NJ: Humana Press), 45–56. doi: 10.1007/978-1-62703-348-0_5

Linta, L., Stockmann, M., Kleinhans, K. N., Böckers, A., Storch, A., Zaehres, H., et al. (2012). Rat Embryonic Fibroblasts Improve Reprogramming of Human Keratinocytes into Induced Pluripotent Stem Cells. *Stem Cells Dev.* 21, 965–976. doi: 10.1089/scd.2011.0026

Liu, J., and Seol, D.-W. (2020). Helper virus-free gutless adenovirus (HF-GLAd): a new platform for gene therapy. *BMB Rep.* 53, 565–575. doi: 10.5483/BMBRep.2020.53.11.185

Liu, Y., Zheng, Y., Li, S., Xue, H., Schmitt, K., Hergenroeder, G. W., et al. (2017). Human neural progenitors derived from integration-free iPSCs for SCI therapy. *Stem Cell Res.* 19, 55–64. doi: 10.1016/j.scr.2017.01.004

Loh, Y.-H., Hartung, O., Li, H., Guo, C., Sahalie, J. M., Manos, P. D., et al. (2010). Reprogramming of T Cells from Human Peripheral Blood. *Cell Stem Cell* 7, 15–19. doi: 10.1016/j.stem.2010.06.004

Mátés, L., Chuah, M. K. L., Belay, E., Jerchow, B., Manoj, N., Acosta-Sanchez, A., et al. (2009). Molecular evolution of a novel hyperactive Sleeping Beauty transposase enables robust stable gene transfer in vertebrates. *Nat. Genet.* 41, 753–761. doi: 10.1038/ng.343

Nakayama, C., Fujita, Y., Matsumura, W., Ujiie, I., Takashima, S., Shinkuma, S., et al. (2018). The development of induced pluripotent stem cell-derived mesenchymal stem/stromal cells from normal human and RDEB epidermal keratinocytes. *J. Dermatol. Sci.* 91, 301–310. doi: 10.1016/j.jdermsci.2018.06.004

Narayanavari, S. A., and Izsvák, Z. (2017). Sleeping Beauty transposon vectors for therapeutic applications: advances and challenges. *Cell Gene Ther. Insights* 3, 131–158. doi: 10.18609/cgti.2017.014

Nishino, K., Toyoda, M., Yamazaki-Inoue, M., Fukawatase, Y., Chikazawa, E., Sakaguchi, H., et al. (2011). DNA Methylation Dynamics in Human Induced Pluripotent Stem Cells over Time. *PLoS Genet.* 7, e1002085. doi: 10.1371/journal.pgen.1002085

Nishino, K., Arai, Y., Takasawa, K., Toyoda, M., Yamazaki-Inoue, M., Sugawara, T., et al. (2018). Epigenetic-scale comparison of human iPSCs generated by retrovirus, Sendai virus or episomal vectors. *Regen. Ther.* 9, 71–78. doi: 10.1016/j.reth.2018.08.002

Nishio, M., and Saeki, K. (2014). “Differentiation of Human Pluripotent Stem Cells into Highly Functional Classical Brown Adipocytes,” in *Methods in Enzymology*, (Elsevier), 177–197. doi: 10.1016/B978-0-12-411619-1.00010-0

Ohmine, S., Dietz, A. B., Deeds, M. C., Hartjes, K. A., Miller, D. R., Thatava, T., et al. (2011). Induced pluripotent stem cells from GMP-grade hematopoietic progenitor cells and mononuclear myeloid cells. *Stem Cell Res. Ther.* 2, 46. doi: 10.1186/scrt87

Okumura, T., Horie, Y., Lai, C.-Y., Lin, H.-T., Shoda, H., Natsumoto, B., et al. (2019). Robust and highly efficient hiPSC generation from patient non-mobilized peripheral blood-derived CD34+ cells using the auto-erasable Sendai virus vector. *Stem Cell Res. Ther.* 10, 185. doi: 10.1186/s13287-019-1273-2

Poleganov, M. A., Eminli, S., Beissert, T., Herz, S., Moon, J.-I., Goldmann, J., et al. (2015). Efficient Reprogramming of Human Fibroblasts and Blood-Derived Endothelial Progenitor Cells Using Nonmodified RNA for Reprogramming and Immune Evasion. *Hum. Gene Ther.* 26, 751–766. doi: 10.1089/hum.2015.045

Poon, M.-W., He, J., Fang, X., Zhang, Z., Wang, W., Wang, J., et al. (2015). Human Ocular Epithelial Cells Endogenously Expressing SOX2 and OCT4 Yield High Efficiency of Pluripotency Reprogramming. *PLOS ONE* 10, e0131288. doi: 10.1371/journal.pone.0131288

Qu, X., Liu, T., Song, K., Li, X., and Ge, D. (2012). Induced Pluripotent Stem Cells Generated from Human Adipose-Derived Stem Cells Using a Non-Viral Polycistronic Plasmid in Feeder-Free Conditions. *PLoS ONE* 7, e48161. doi: 10.1371/journal.pone.0048161

Schlaeger, T. M., Daheron, L., Brickler, T. R., Entwisle, S., Chan, K., Cianci, A., et al. (2015). A comparison of non-integrating reprogramming methods. *Nat. Biotechnol.* 33, 58–63. doi: 10.1038/nbt.3070

Sharma, A., Mücke, M., and Seidman, C. E. (2018). Human Induced Pluripotent Stem Cell Production and Expansion from Blood using a Non‐Integrating Viral Reprogramming Vector. *Curr. Protoc. Mol. Biol.* 122, e58. doi: 10.1002/cpmb.58

Si-Tayeb, K., Noto, F. K., Sepac, A., Sedlic, F., Bosnjak, Z. J., Lough, J. W., et al. (2010). Generation of human induced pluripotent stem cells by simple transient transfection of plasmid DNA encoding reprogramming factors. BMC Dev Biol 10, 81. doi: 10.1186/1471-213X-10-81

Su, R.-J., Baylink, D. J., Neises, A., Kiroyan, J. B., Meng, X., Payne, K. J., et al. (2013). Efficient Generation of Integration-Free iPS Cells from Human Adult Peripheral Blood Using BCL-XL Together with Yamanaka Factors. *PLoS ONE* 8, e64496. doi: 10.1371/journal.pone.0064496

Sugii, S., Kida, Y., Berggren, W. T., and Evans, R. M. (2011). Feeder-dependent and feeder-independent iPS cell derivation from human and mouse adipose stem cells. *Nat. Protoc.* 6, 346–358. doi: 10.1038/nprot.2010.199

Takayama, N., Nishimura, S., Nakamura, S., Shimizu, T., Ohnishi, R., Endo, H., et al. (2010). Transient activation of *c-MYC* expression is critical for efficient platelet generation from human induced pluripotent stem cells. *J. Exp. Med.* 207, 2817–2830. doi: 10.1084/jem.20100844

Tan, X., Dai, Q., Guo, T., Xu, J., and Dai, Q. (2018). Efficient generation of transgene- and feeder-free induced pluripotent stem cells from human dental mesenchymal stem cells and their chemically defined differentiation into cardiomyocytes. *Biochem. Biophys. Res. Commun.* 495, 2490–2497. doi: 10.1016/j.bbrc.2017.12.007

Trokovic, R., Weltner, J., Nishimura, K., Ohtaka, M., Nakanishi, M., Salomaa, V., et al. (2014). Advanced Feeder-Free Generation of Induced Pluripotent Stem Cells Directly From Blood Cells. *Stem Cells Transl. Med.* 3, 1402–1409. doi: 10.5966/sctm.2014-0113

Warren, L., Manos, P. D., Ahfeldt, T., Loh, Y.-H., Li, H., Lau, F., et al. (2010). Highly Efficient Reprogramming to Pluripotency and Directed Differentiation of Human Cells with Synthetic Modified mRNA. *Cell Stem Cell* 7, 618–630. doi: 10.1016/j.stem.2010.08.012

Wen, W., Zhang, J.-P., Xu, J., Su, R. J., Neises, A., Ji, G.-Z., et al. (2016). Enhanced Generation of Integration-free iPSCs from Human Adult Peripheral Blood Mononuclear Cells with an Optimal Combination of Episomal Vectors. *Stem Cell Rep.* 6, 873–884. doi: 10.1016/j.stemcr.2016.04.005

Woltjen, K., Hämäläinen, R., Kibschull, M., Mileikovsky, M., and Nagy, A. (2011). “Transgene-Free Production of Pluripotent Stem Cells Using piggyBac Transposons,” in *Human Pluripotent Stem Cells*, eds. P. H. Schwartz and R. L. Wesselschmidt (Totowa, NJ: Humana Press), 87–103. doi: 10.1007/978-1-61779-201-4_7

Wu, S. C.-Y., Meir, Y.-J. J., Coates, C. J., Handler, A. M., Pelczar, P., Moisyadi, S., et al. (2006). *piggyBac* is a flexible and highly active transposon as compared to *Sleeping Beauty* , *Tol2* , and *Mos1* in mammalian cells. *Proc. Natl. Acad. Sci.* 103, 15008–15013. doi: 10.1073/pnas.0606979103
