# Supplementary material for: A comprehensive analysis of induced pluripotent stem cell (iPSC) production and applications
Source: Front Cell Dev Biol. 2025 May 8;13:1593207. doi: 10.3389/fcell.2025.1593207 (PMC12095295; doi:10.3389/fcell.2025.1593207)
Supplement: Supplementary file 2 [file Table2.docx]

**Table S2. Outcomes of iPSCs-based therapies by therapeutic area**

| **Therapeutic area** | **Cell origin** | **Reprogramming method** | **Purpose** | **Disease** | **Animal model** | **Study design** | **Outcome** | **Reference** |
| --- | --- | --- | --- | --- | --- | --- | --- | --- |
| **Ocular diseases** | Human fibroblasts | Lentivirus transduction | Retinal pigment epithelium (RPE) replacement therapy | Age-related macular degeneration (AMD) | Immunodeficient Royal College of Surgeons (RCS) rat | The density of each implant was kept at approximately 2700 cells/membrane. Only the left eyes were used for transplantation | The transplants remained as a monolayer, expressed RPE-specific markers, performed phagocytic function, and contributed to vision preservation. At 11-months post-implantation, RPE survival was observed in only 50% of the eyes that were concomitant with vision preservation. | (Rajendran Nair et al., 2021) |
|  | Human PBMCs | Sendai virus, expressing the Yamanaka factors Oct4, Sox2, Klf4, and c-Myc, and utilizing the CytoTune-iPS 2.0 Sendai Reprogramming Kit | iMSC Transplantation in Rotenone-Injured Eyes | Rotenone-injured eyes | C57BL/6JNarl mice | One day rotenone-induced LHON animal model induction, 2 × 10^5^ iMSCs into the LHON animal model’s eyes via an intravitreal injection. |  | (Tsai E.-T. et al., 2023) |
|  | Human CD34+ peripheral blood cells | Set of plasmids | Clinical-grade AMD patient–specific iRPE patch | Age-related macular degeneration (AMD) | Immunocompromised (Crl:NIH-Foxn1rnu) rat | AMD iRPE patches (100,000 or 10,000 cells) were transplanted in the subretinal space of rat eyes. | Fundus infrared imaging and optical coherence tomography (OCT) 10 weeks after surgery confirmed successful integration of the patch under the host retina. | (Sharma R. et al., 2019) |
|  | Mouse fibroblasts | Retrovirus (mouse OKSM) | Mouse iPSC-TM (trabecular meshwork) | Glaucoma | Tg-MYOCY437H mice | Therefore, 50,000 purified iPSC-TM were injected into the anterior chamber of 4-mo-old Tg-MYOCY437H mice in a volume of 3 µL PBS (n = 22). | Transplanted iPSC-TM survive in the TM, but the most pronounced effect of transplantation is a robust proliferative response of endogenous TM cells. | (Zhu W. et al., 2016) |
|  | Mouse fibroblasts | Retrovirus (Oct-3/4, Sox2, Klf4, and c-Myc) | iPSC-TM (trabecular meshwork) | Glaucoma | C57BL/6 mice | 50,000 iPSC-TM cells transfected with either Cx43 shRNA or scrambled shRNA were resuspended in 3 µL 1 × PBS (Gibco) and injected into the anterior chamber. | Quantification of overall TM cell density indicated that transplantation of iPSC-TM cells expressing scrambled shRNA significantly increases the overall number of TM cells in Ad5-MYOCY437H-EGFP mice. | (Sui et al., 2021) |
|  | N/A | N/A | iPSC-derived trabecular meshwork (miPSC-TM) | Glaucoma | C57BL/6J mice | 75,000 miPSC-TM labeled with PSC NPs (35 μg/mL) were intracamerally injected into the eyes of mice. | The usage of nanoparticle improves the delivery accuracy of the transplanted cells in live animals and also benefits the dual-model tracking in the long term. More importantly, the use of the magnet triggers a temporary enhancement in the effectiveness of cell-based therapy in alleviating the pathologies associated with glaucoma. | (Wang X. et al., 2022) |
|  | N/A | N/A | Retinal pigment epithelium derived from induced pluripotent stem cells (iPS-RPE cells) | N/A | Cynomolgus monkey | iPS-RPE cells (single-cell suspensions: 1–2 × 10^5^ cells/eye) were transplanted into the subretinal space. | An inhibitor of Rho-associated protein kinase (ROCK) effected in vivo on the transplantation of iPS-RPE cell suspensions and also increased the viability of the transplant without showing obvious retinal toxicity in human iPS-RPE transplantation. | (Ishida et al., 2021). |
|  | Retinal organoid | Electroporate piggyBac and transposase vectors into PGP1 cells in suspension | Human photoreceptors | Blinding diseases | Cpfl1-mutant mice | 150,000 cells/μL injected into the subretinal space of host eyes. | iPSC-derived human photoreceptors interacted readily with a partially degenerated retina. | (Gasparini et al., 2022) |
| **Nervous system disorders** | Human PBMCs | Episomal plasmid vector (Oct3/4 , Sox2, Klf4, L-Myc, LIN28, mp53DD и EBNA1) | iPSC-derived DA progenitors (DAPs) | Parkinson’s disease (PD) | 6-hydroxydopamine (6-OHDA)-lesioned rats | Cell transplantation was performed with the stereotactic injection of 4 × 10^5^ cells into the right striatum. | The DAPs have no tumorigenicity or toxicity and that they improve the abnormal behavior of 6-hydroxydopamine (6-OHDA)-lesioned rats. | (Doi et al., 2020) |
|  | Fibroblasts | The StemMACS iPSC mRNA Reprogramming Kit | Dopaminergic Neuron | PD | RAG2 KO mice (B6.Cg-Rag2tm1.1Cgn/J) | Three weeks after the lesion, 1 µL of cell suspension (100,000 cells/µL) was injected into the substantia nigra pars compacta (SNpc) at the same coordinates used for 6-OHDA injection. | The midbrain DA neurons derived from hiPSCs grafted homotopically into the 6-OHDA lesioned SNpc, sent axons to the targets of mDA neurons. The long-term motor functional recovery after transplantation was correlated to the number of DA neurons within the graft. | (Brot et al., 2022) |
|  | N/A | Episomal plasmid vectors | Induced pluripotent stem cell-derived neural stem/progenitor cells (hiPSC-NS/PCs) | Spinal cord injury (SCI) | Nonobese diabetic severe combined immune deficient (NOD-SCID) mice | hM3Dq-NS/PCs were transplanted into the lesion epicenter of mice. The hM3Dq-NS/PCs neurospheres (5 × 10^5^ cells) were suspended in 2 μL of PBS. | The consecutive and selective chemogenetic stimulation of transplanted hiPSC-NS/PCs enhanced the expression of synapse-related genes and proteins in surrounding host tissues and prevents atrophy of the injured spinal cord, thereby improving locomotor function. | (Kawai et al., 2021) |
|  | Human fibroblasts | oriP/EBNA1-based episomal vectors encoding the human genes Oct4, Sox2, Nanog, LIN28, c-Myc and Klf4. | Human iPSC-derived neural stem cells (NSCs) selected for the expression of specific markers, namely, Lewis X, CXCR4 and beta 1 integrin, and pretreated with neurotrophic factors and apoptosis/necroptosis inhibitors | Spinal muscular atrophy with respiratory distress type 1 (SMARD1) | The nmd mice and in NOD/SCID mice | An intrathecal infusion of the cell suspension (2 μl with 10,000 cells/μl) in intervertebral space. | The cells were detected in the ventral horn of the spinal cord and observed improvements in neuropathological features, particularly preservation of the integrity of the motor unit, that were correlated with amelioration of the SMARD1 disease phenotype in terms of neuromuscular function and lifespan. | (Forotti et al., 2019) |
|  | Human fibroblasts | Sendai virus | Human iPSC-derived microglial (iMG) progenitors | Adult-onset leukoencephalopathy with axonal spheroids and pigmented glia (ALSP) | hFIRE mice | 2 μL of cell suspension in sterile 1× DPBS at 62,500 cells/μL at each injection site. The hematopoietic progenitor cells (HPCs) were transplanted bilaterally into the lateral parietal association cortex and dorsal hippocampus at the following coordinates relative to bregma. | Human microglia fully repopulate the brains of hFIRE mice, transplantation of microglial progenitors prevents the formation of axonal spheroids. Microglia engraftment prevents the development of additional ALSP-related pathologies. | (Chadarevian et al., 2024) |
|  | Human fibroblasts | Sendai virus vectors kit with [reprogramming factors (Oct-3/4, Klf-4, Sox-2, and c-Myc)](https://www.ncbi.nlm.nih.gov/pmc/articles/PMC7363942/#sec4title) | Hippocampal spheroids | Alzheimer’s disease (AD) | RAG-2-deficient mice | A volume of 2 μl of cell suspension was injected at a rate of 0.5 μl/min at the following coordinates (from bregma and brain surface) through drilled holes in the skull | The human cells had survived and integrated in the host hippocampi, 5% of all human cells were still actively dividing. Human iPSC-derived hippocampal spheroids survived in the mouse brain and express neuronal and astrocytic markers. | (Pomeshchik et al., 2023) |
|  | Cord blood | Episomal | Human iPSC‐derived neural precursor cells (iPSC‐NPCs) | Huntington’s disease (HD) | YAC128 transgenic and wild‐type (FVB/N) mice | 4 μl of HLA‐iPSC‐NPCs (100 000 cells/μl) were injected into the striatum of both hemispheres. | HLA‐iPSC‐NPCs improved motor and cognitive function and differentiated into neurons, astrocytes, oligodendrocytes in mice. | (Park et al., 2021) |
|  | N/A | N/A | Neural Progenitor Cells | SCI | NIH nude rats | 1 μL of cell suspension (150,000 cells) was injected into each injured spinal cord. | The grafted iPSC-NPCs were able to integrate into the injured spinal cord and differentiate into neurons and glia and, importantly, significantly improve forelimb functional locomotor recovery. | (Zheng et al., 2022) |
|  | Human PBMCs | CytoTune-iPS Sendai Reprogramming Kit containing Yamanaka factors | iPSC-derived motor neuron progenitor cells (iMNP) and iPSC-derived motor neuron (iMN) | SCI | Sprague-Dawley (SD) rats | The stepwise cell therapy by injecting iMNP six-weeks after MSC transplantation. Preemptive cell therapy was performed with 1 × 10^6^ MSCs via intravenous injection at 24 h and one-week post injury. At six-weeks post injury, the lesional and injured sites were re-exposed, and 1 × 10^6^ iMNPs were transplanted. | Stepwise combined cell therapy promoted mature motor neuron differentiation and axonal regeneration at the lesional site. In addition, stepwise combined cell therapy improved behavioral recovery and was more effective than single cell therapy alone. | (Kim J.-W. et al., 2024) |
|  | Human urine epithelial cells | N/A | Urine derived iPSCs derived patient-specific neural progenitor cells (UiPSC-NPCs) | SCI | Sprague-Dawley rats | UiPSC-NPCs were transplanted stereotaxically into two sites (2.5 μL / site, 1 × 10^5^ cells / μL). | Transplantation of UiPSC-NPCs showed little effect on microglia activation at the lesion epicenter. The UiPSC-NPCs transplanted at the subacute phase of SCI showed a beneficial effect on tissue repairing. | (Liu A. et al., 2020) |
|  | N/A | N/A | Neural progenitor cells (NPCs) | Post-traumatic stress disorder (PTSD) | Male Sprague-Dawley rats | 1 × 10^6^ iPSC-NPCs were transplanted into the hippocampus site by intracranial positioning. | Transplantation of IPSC-NPCs into PTSD rats promoted regeneration and motor function recovery in PTSD model. | (Liu Q. et al., 2021) |
|  | N/A | N/A | hiPSC-NS/PCs | SCI | NOD-SCID mice | 5.0 × 105/2 μL hiPSC-NS/PCs into 2 points on the rostral and caudal sides of the injured epicenter. | Rehabilitative training promoted the survival rate and neuronal differentiation of transplanted hiPSC-NS/PCs. | (Shibata et al., 2023) |
| **Cancer** | A child’s foreskin “somatic cells” | LV transduction | iPSC-derived natural killer (iNK) cells for immunotherapy | Ovarian cancer | NOD.Cg-Prkdcscid Il2rgtm1Wjl/SzJ (NSG) mice | 1 × 10^7^ iNK cells were injected i.p. into tumor-bearing mice along with 5 × 10^4^ U IL-2, or mice were left untreated. IL-2 was then administered i.p. twice weekly for the next two weeks. For experiments testing iNK cells in combination with T cells and checkpoint inhibitory receptor blockade, groups of mice received i.p. injections of 5 × 10^6^ iNK cells, 2.5 × 10^6^ CD3+ T cells for T cell recruitment experiments, with or without 100 mg pembrolizumab per mouse along with the first injection of IL-2. | iNK cells recruited T cells and cooperated with T cells and anti-PD-1 antibody, further enhancing inflammatory cytokine production and tumor lysis. | (Cichocki et al., 2020) |
|  | Mouse Embryonic Fibroblasts (MEFs) | Retrovirus transduction | Functional and highly pure-induced macrophage (iMac) | Malignant cancers | BALB/c mice, NCG mice | Injection of 1 × 10^6^ iMac (from mice of the same strain) via the tail vein for BALB/c, injection of 2 × 10^6^ iMac cells by tail vein for NCG mice. | iMac cells displayed significantly increased in vivo persistence and inhibition of tumor progression in leukemia, breast cancer, and patient-derived tumor xenograft models. | (Li S. et al., 2024) |
|  | C57BL/6Jmurine iPSCs | N/A | Murine iPSCs | Pancreatic ductal adenocarcinoma (PDAC) | C57BL/6J | 2 × 10^6^ autologous murine iPSCs were irradiated at 6,000 rads before injection. Irradiated iPSCs were suspended in 5 μM CpG ODN1826 in PBS by subcutaneous injection of the vaccine in the flanks of the mice. | The iPSC-based cancer vaccine prevents tumor growth in pancreatic cancer and induces cytotoxic antitumor T cell and B cell responses. | (Ouyang et al., 2021) |
|  | Mouse fibroblasts | Sendai reprograming kit | Neoantigen-augmented iPSCs (NA-iPSCs) | Colorectal cancer (CRC) and triple-negative breast cancer (TNBC) | BALB/c | 2 × 10^6^ autologous murine iPSCs were irradiated (50 Gy) and suspended in PBS and subcutaneous immunized in the flanks of the mice. | After administration of NA-iPSCs cancer vaccine and radiotherapy, ~60% of tumor-bearing mice achieved a complete response in microsatellite-stable CRC model. The therapeutic efficacy of NA-iPSCs engineered by mouse TNBC neoantigens was also observed in the syngeneic immunocompetent TNBC mouse model. | (Huang K. C.-Y. et al., 2024) |
|  | N/A | N/A | Murine induced pluripotent stem cells (miPSCs) | Melanoma | C57BL/6 mice | The miPSC/H6 vaccine consisted of 1 × 10^6^ miPSCs, admixed with 5 × 105 B16H6 cells. Mice were immunized subcutaneously twice. | Mice treated with iPSC vaccine demonstrated increased activation of the immune response in the vaccination site and tumor microenvironment. | (Gąbka-Buszek et al., 2020) |
|  | N/A | N/A | Syngeneic iPSC | Melanoma | C57BL/6 mice | 2 × 10^6^ irradiated (60Gy) iPSC admixed with 500 pmol CpG (Toll-like receptor (TLR) 9 agonist as an adjuvant). | Treatment of mice significantly reduced the number of lung metastases. | (Hundt et al., 2021) |
| **Diseases of internal organs** | N/A | N/A | Endocrinologically active pancreatic islet cells | Diabetes | NSG-SGM3 mice | One thousand islet clusters were resuspended in 60 μl of sterile saline and injected intramuscularly into the hindlimb muscle. | iPSC-derived cells survived in immunocompetent, allogeneic diabetic humanized mice for 4 weeks and ameliorated diabetes. | (Hu X. et al., 2024) |
|  | Human PSCs | N/A | Stem cell-derived pancreatic β (SC-β) cells | Diabetes | NOD.Cg-Prkdcscid Il2rgtm1Wjl/SzJ mice | 5 × 10^6^ islet cells under the kidney capsule. | Transplantation of islet-sized aggregates of SC-β cells reversed severe preexisting diabetes in mice at a rate close to that of human islets and maintained normoglycemia for at least 9 months. | - (Hogrebe et al., 2020) |
|  | Human iPSC UC | N/A | Hepatobiliary organoids (HBOs) | End-stage liver disease | Cynomolgus monkeys | Injection of HBOs (total 2×10^6^ cells/kg) at 5 transplantation sites under the liver capsule. | HBO transplantation could safely and effectively improve hepatoprotection effects by antiapoptotic and antifibrotic agents. | (Li H. et al., 2024) |
|  | N/A | Simplicon® RNA Reprogramming Kit (OKSG) | iPSC-derived kidney organoids | Kidney disease | NOD.Cg-PrkdcscidIl2rgtm1Wjl/SzJ | Kidneys were exteriorized via flank incisions and bisected kidney organoids were transplanted under the renal capsule through a small incision. | The modulating HLA class II signaling in iPSC-derived kidney organoids will be necessary to prevent rejection following transplantation. | (Gaykema et al., 2024) |
|  | Human fibroblasts | Sendai Reprogramming Kit | Podocytes (iPSC-PODs) | Chronic kidney disease | C57BL/6J mice | A left intrarenal injection of 1 × 10^5^ iPSC-PODs in 10 μL PBS or a control injection of PBS. | Transplanted iPSC–PODs can survive in recipient newborn mouse kidneys due to the immature and immunoprivileged nature of the developing postnatal kidneys. | (Lau et al., 2020) |
|  | N/A | N/A | iPSC-hepatocytes (REPROCELL) | N/A | NOD/SCID mice | 1×10^6^ iPSC-hepatocytes were encapsulated into a core-shell microfiber formed. The fibers were transplanted into their abdominal cavities using the spatulas. | Three days after transplantation of the microfibers into the abdominal cavity, human albumin (characteristics of iPSC-hepatocytes) was detected in the peripheral blood of the transplanted mice. | (Nagata et al., 2020) |
| **Muscle dysfunctions and cardiomyopathies** | Human iPSCs | CytoTune-iPS Sendai reprogramming kit | Skeletal muscle myoblasts (iMyoblasts) | Muscular dystrophies | NOD.Cg-PrkdcscidIL2rγtmiWjl /SzJ mice | 1×10^6^ bMyoblasts (myoblasts from adult muscle biopsy myoblasts) or iMyoblasts were resuspended in 50 μl 1 mg/ml laminin in PBS and injected bilaterally into the body of tibialis anterior muscles. | iMyoblasts efficiently xenoengrafted into irradiated and injured mouse muscle where they demonstrate their regulatory plasticity for adult muscle maturation in response to signals in the host muscle. | (Guo et al., 2022) |
|  | The human iPSC lines Ff-WJ14s01 (from cord blood cells) and 414C2 (from human dermal fibroblast). | Episomal vectors | Muscle stem cells (MuSCs) | Duchenne muscular dystrophy (DMD) | The NOG-mdx mouse strain (NOD.Cg-Prkdcscid Dmdmdx il2rgtm1Sug/Jic) | Freshly sorted cells were resuspended, and 1×10^4^ cells per injection site (primary satellite cells) or 1×10^5^ cells per injection site (GFP-transgenic Hu5/KD3 and iMuSCs) were injected. The injection volume per site was 10 μL. | A method for cell transplantation into mouse diaphragm was established and showed that an injectable hyaluronic acid-gelatin solution enables the engraftment of iMuSCs in the diaphragm. | (Miura et al., 2022) |
|  | N/A | N/A | Human-induced pluripotent stem cell-derived cardiomyocytes (iPSC-CMs) | Myocardial infarction | Sprague-Dawley rats | One intramyocardial injection of 5% albumin solution with or without 1 × 10^7^ human iPSC-CMs 10 days after undergoing left anterior descending coronary artery ligation. Cyclosporine A and methylprednisolone were administered before iPSC-CM injection and until the rats were killed to prevent graft rejection. | iPSC-CM injection improves cardiac function. | (Guan X. et al., 2020) |
|  | HEK293 | Transfection lenti-α-MHC/CDH2 plasmid with MISSION LV packaging mix | Human-induced pluripotent stem cell-derived cardiomyocytes (hiPSC-CMs) | Myocardial infarction | NOD/SCID gamma mice | WT-hiPSC-CMs or CDH2-hiPSC-CMs (3 × 10^5^ CMs/mouse) were delivered into the infarct and border zones by means of three intra-myocardial injections (equal volume, 1 × 10^5^ CMs/site). | The measurements of cardiac functions were significantly higher in CDH2-hiPSC-CMs treated group than in WT-hiPSC-CMs treated group. | (Lou et al., 2020) |
|  | C57BL/6 (B6) (CLEA) mouse embryonic fibroblasts | N/A | Induced pluripotent stem cell-derived cardiomyocyte (iPSC-CM) | Heart failure | BALB/c mice | Intramuscular injection of allogeneic iPSC-CM sheets with 200 µl of PBS alone (CM) or 5 × 10^6^ MSCs in 200 µl of PBS (CM + MSC) at the site of iPSC-CM sheet transplant. | The co-transplantation of MSCs might control immune rejection against allogeneic iPSC-CMs after in vivo transplantation through the Treg induction and direct cell–cell contact. | (Yoshida S. et al., 2020) |
| **Skin, cartilage, bones, joints** | PBMC | Episomal vectors | iPSC‐derived chondrocytes in gelatin methacryloyl (GelMA) hydrogel | Full‐thickness articular cartilage defects | Nude mice | iPSC-derived chondrocytes were encapsulated in GelMA hydrogel at low (1 × 10^7^ ml^−1^) and high (2 × 10^7^ ml^−1^) density. After cell-laden or cell-free constructs were implanted in the subcutaneous pockets on the back of mice. | Direct ectopic implantation of iPSC-derived chondrocyte-laden GelMA, without in vitro priming, generated hyaline cartilage-like tissue. | (Agten et al., 2022) |
|  | N/A | N/A | The cynomolgus monkey iPSCs (cyiPSCs) derived cartilage organoid (cyiPS-Cart) particles | Osteoarthritis | Cynomolgus monkeys | After chondrogenic differentiation, the cells were transferred into suspension culture to form cartilaginous particles 1–3 mm in diameter. | cyiPS-Cart survived and directly contributed to hyaline cartilage-rich repaired tissue in chondral defects. | (Abe et al., 2023) |
|  | Primary endothelial colony forming cells (ECFCs), skin fibroblasts (FBs) and keratinocytes (KCs) | Sendai virus | Endothelial cells (hiPSC-ECs), fibroblasts (hiPSC-FBs) and keratinocytes (hiPSC-KCs) | Skin damage | NOD.Cg-Prkdcscid Il2rgtm1WjI/SzJ mice | hiPSC-derived single-cell suspensions consisting of 6 x 10^6^ hiPSC-KCs, 3 x 10^6^ hiPSC-FBs and 3 x 10^6^ hiPSC-ECs were grafted. | iPSC-derived skin cell-suspension after liquid-transplantation, healed deep wounds of mice after 2 weeks. | (Ebner-Peking et al., 2021) |
|  | C57BL/6 MEFs | Retroviral vectors encoding murine Oct4, Sox2, Klf4, and c-Myc | Osteoblasts | Critical-size bone defect | C57BL/6 mice | For transplantation model the scaffolds were seeded with 2.5 × 10^6^ iPSCs directly onto the scaffold and incubated in osteogenic medium for 3 days. | Transplantation of iPSC-seeded PLGA/aCaP scaffolds may improve bone regeneration in critical-size bone defects in mice. | (Kessler et al., 2024) |
|  | Human skin cells | Retroviral transduction | iPSC-derived mesenchymal stem cells (iPSC-MSCs) | Narrowing and stenosis of the upper airway | New Zealand white rabbits | 3D-printed tracheal scaffolds were prepared and seeded primary bronchial epithelial cells. Dissociated hiPSC-derived MSCs were suspended at a density of 1 × 10^6^ cells/mL in Matrigel, and the suspension was then applied in an even coating on the outer surfaces of the scaffolds. The scaffolds pre-incubated in growth media for 2 days using the bioreactor system. | 3D-printed nanofiber artificial trachea combined with various cell types can regenerate into a functional, cartilaginous, epithelialized airway. | (Kim I. G. et al., 2020) |
|  | Mouse fibroblasts | Retroviral transduction | Lubricin-expressing cells | Osteoarthritis and synovitis | SCID mice | Injection of 1 × 10^4^ cells in the paratenon surrounding the Achilles tendons and knee joints. | The cells’ survival and lubricin expression in vivo were confirmed. | (Satake et al., 2022) |
| **Other studies** | Human fibroblasts | LV transduction (POU5F1, Sox2, Klf4, and c-Myc) | Kidney Organoid | N/A | BALB/c IL2Ry−/−RAG2−/− immune deficient mice | Organoids were implanted with 50 µL Geltrex. One month after organoid implantation, 1 × 10^7^ PBMC were administered via intraperitoneal injection. | Subcutaneous implantation of kidney organoids in immune-deficient mice followed by adoptive transfer of human PBMC led to the invasion of diverse T-cell subsets. This study characterized the interaction between immune cells and kidney organoids. | (Shankar et al., 2024) |
|  | Human PBMCs or BMMCs | LV vector or the cocktail CytoTune-iPS 2.0 Sendai reprogramming kit | Acute myeloid leukemia (AML) cells reprogramming into induced pluripotent stem cell (AML-iPSCs) lines | AML | NSG and NSGS mice | Injection of 1 × 10^6^ cells via the tail vein. | These AML-iPSCs retain genetic fidelity and produce transplantable hematopoietic cells with hallmark phenotypic leukemic features. | (Kotini et al., 2023) |
|  | Human PBMC | Non-integrating vector with genes (POU5F1, Sox2, Klf4, c-Myc, Lin28) | iHPCs (hematopoietic progenitor cells) | Familial Alzheimer’s Disease (FAD) | 5xFAD-MITRG mice | Mice received 1 μL of iHPCs suspended in sterile at 62.5K cells/μL at each injection site (8 sites) totaling 500K cells/pup. | The engrafted iHPCs had differentiated into microglia. Transplanted iHPCs acquired morphologies and phenotypic signatures in a niche-dependent manner. | (Hasselmann et al., 2019) |
|  | Porcine iPSCs | Retrovirus transduction (Oct4, Klf4, Sox2 and c-Myc) | piPSC-derived endothelial cells (piPSC-ECs) | Cardiovascular drug screening, model for the mechanistic studies on EC differentiation and endothelial dysfunction | NOD/SCID mice | A total of 1 × 10^7^ cells were injected subcutaneously into the abdominal flanks of mice. | The above results showed that the derived piPSC-ECs had angiogenic function both in vivo and in vitro, indicating their great potential in fundamental research and pre-clinical experiments. | (Li X. et al., 2021) |
|  | Human iPSC | N/A | iPSC-derived endothelial cells (iECs) | This modular design could contribute to the advancement of allogeneic cell therapeutics | NSG-SGM3 mice | A total of 5 × 10^4^ wild type or engineered iECs were injected subcutaneously into humanized NSG-SGM3 mice mixed together with 1 × 10^6^ human NK cells. | A new class of agonistic immune checkpoint engagers protects human leukocyte antigen (HLA)-depleted iECs from innate immune cells were presented. | (Gravina et al., 2023) |
|  | Human PBMC | Episomal plasmids | iPSC-derived hematopoietic progenitor cells (HPC) | in vivo model for neuro human immunodeficiency virus type-1 (HIV-1) | Neonatal immunocompromised mice | Injection of 400–500k HPCs at four cranial surface coordinates at two different depth, at 6–10 weeks mice were intraperitoneally injected with human PBMCs. | The mice model for investigating the genetic mechanisms governing central nervous system HIV-1 infection and latency at a single-cell level were presented. | (Min et al., 2023) |
|  | Mouse ovarian granulosa cell | N/A | iPSC-derived mouse ovarian granulosa cell (mGriPSCs) | Premature ovarian insufficiency (POI) | B6.Cg-Foxn1^nu^/J nude mice | An intramuscular injections of 2 x 10^6^ unsorted mGriPSCs, sorted mGriPSCs in 100 μLPBS or vehicle into the left thigh. | Endocrine function and fertility were restored in mice pretreated with gonadotoxic alkylating agents via orthotopic transplantation of differentiated iPSCs. | (Elias et al., 2023) |
|  | Human iPSCs | N/A | N/A | NuRabbits as the recipient animals in xenotransplantation experiments using human iPCSs | FOXN1 mutant nude rabbits (NuRabbits) | One intramuscular injections of 1 × 10^6^ iPSCs in each hindleg | NuRabbits as a new member of the immunodeficient animal model family were presented. | (Song et al., 2021) |
|  | Human iPSCs | N/A | iPSC–derived platelet product (iPSC-PLTs) | Thrombocytopenia | Rabbit | 1×10^10^ platelets per 2.5 kg body weight were injected into the left ear marginal vein | A complete system for the GMP-based production of iPSC-PLTs. | (Sugimoto et al., 2022) |

References:

Chadarevian, J. P., Hasselmann, J., Lahian, A., Capocchi, J. K., Escobar, A., Lim, T. E., et al. (2024). Therapeutic potential of human microglia transplantation in a chimeric model of CSF1R-related leukoencephalopathy. *Neuron* 112, 2686-2707.e8. doi: 10.1016/j.neuron.2024.05.023

Forotti, G., Nizzardo, M., Bucchia, M., Ramirez, A., Trombetta, E., Gatti, S., et al. (2019). CSF transplantation of a specific iPSC-derived neural stem cell subpopulation ameliorates the disease phenotype in a mouse model of spinal muscular atrophy with respiratory distress type 1. *Exp. Neurol.* 321, 113041. doi: 10.1016/j.expneurol.2019.113041

Gasparini, S. J., Tessmer, K., Reh, M., Wieneke, S., Carido, M., Völkner, M., et al. (2022). Transplanted human cones incorporate into the retina and function in a murine cone degeneration model. *J. Clin. Invest.* 132, e154619. doi: 10.1172/JCI154619

Liu, Q., Zhang, L., and Zhang, J. (2021). Induced pluripotent stem cell-derived neural progenitor cell transplantation promotes regeneration and functional recovery after post-traumatic stress disorder in rats. *Biomed. Pharmacother.* 133, 110981. doi: 10.1016/j.biopha.2020.110981

Park, H. J., Jeon, J., Choi, J., Kim, J. Y., Kim, H. S., Huh, J. Y., et al. (2021). Human iPSC‐derived neural precursor cells differentiate into multiple cell types to delay disease progression following transplantation into YAC128 Huntington’s disease mouse model. *Cell Prolif.* 54, e13082. doi: 10.1111/cpr.13082

Sharma, R., Khristov, V., Rising, A., Jha, B. S., Dejene, R., Hotaling, N., et al. (2019). Clinical-grade stem cell–derived retinal pigment epithelium patch rescues retinal degeneration in rodents and pigs. *Sci. Transl. Med.* 11, eaat5580. doi: 10.1126/scitranslmed.aat5580

Tsai, E.-T., Peng, S.-Y., Wu, Y.-R., Lin, T.-C., Chen, C.-Y., Liu, Y.-H., et al. (2023). HLA-Homozygous iPSC-Derived Mesenchymal Stem Cells Rescue Rotenone-Induced Experimental Leber’s Hereditary Optic Neuropathy-like Models In Vitro and In Vivo. *Cells* 12, 2617. doi: 10.3390/cells12222617

Zhu, W., Gramlich, O. W., Laboissonniere, L., Jain, A., Sheffield, V. C., Trimarchi, J. M., et al. (2016). Transplantation of iPSC-derived TM cells rescues glaucoma phenotypes in vivo. *Proc. Natl. Acad. Sci.* 113, E3492-3500. doi: 10.1073/pnas.1604153113
