# Supplementary material for: A comprehensive analysis of induced pluripotent stem cell (iPSC) production and applications
Source: Front Cell Dev Biol. 2025 May 8;13:1593207. doi: 10.3389/fcell.2025.1593207 (PMC12095295; doi:10.3389/fcell.2025.1593207)
Supplement: Supplementary file 3 [file Table3.docx]

**Table S3. iPSC-based clinical trials**

| **Trial ID** | **Title** | **Status** | **Description** | **Phase** | **Type of cell** | **Condition** | **Country** | **Enrolment** |
| --- | --- | --- | --- | --- | --- | --- | --- | --- |
| NCT02923375 | A Study of CYP-001 for the Treatment of Steroid-Resistant Acute Graft Versus Host Disease | Completed |  | 1 | Allogenic | Steroid-Resistant Acute Graft Versus Host Disease | Australia, UK | 16 |
| NCT03696628 | Modeling and Pharmacological Targeting of Genetic Cardiomyopathy in Children *Via* Cardiomyocytes Derived From Induced Pluripotent Stem Cells (DMDstem) (DMDstem) | Completed |  | N/A | Autologous | Cardiomyopathy | France | 24 |
| NCT04339764 | Autologous Transplantation of Induced Pluripotent Stem Cell-Derived Retinal Pigment Epithelium for Geographic Atrophy Associated With Age-Related Macular Degeneration | Recruiting | To test the safety of putting cells inside the eye. | 1,2 | Autologous | Dry Age-Related Macular Degeneration  Geographic Atrophy | USA | 20 |
| NCT04537351 | The MEseNchymal coviD-19 Trial: MSCs in Adults With Respiratory Failure Due to COVID-19 or Another Underlying Cause | Completed |  | 1 | Allogenic | Covid19, Acute Respiratory Distress Syndrome | Australia | 14 |
| NCT04945018 | A Study of iPS Cell-derived Cardiomyocyte Spheroids (HS-001) in Patients With Heart Failure (LAPiS Study) | Recruiting | To evaluate the safety and efficacy of HS-001 transplanted into severe heart failure patients for 26 weeks after transplantation. | 1,2 | Allogenic | Heart Failure, Ischemic Heart Disease | Japan | 10 |
| NCT04982081 | Treating Congestive HF With hiPSC-CMs Through Endocardial Injection | Unknown |  | 1 | Allogenic | Cardiovascular Diseases, Congestive Heart Failure, Dilated Cardiomyopathy | China | 20 |
| NCT05445063 | Safety and Efficacy of Autologous Transplantation of iPSC-RPE in the Treatment of Macular Degeneration | Recruiting | To test the efficacy and safety of RPE transplants to treat macular degeneration. | 1 | Autologous | Macular Degeneration | China | 10 |
| NCT05643638 | A Study of CYP-001 in Combination With Corticosteroids in Adults With High-risk aGvHD | Recruiting | To investigate the efficacy and safety of CYP-001 in combination with corticosteroids vs corticosteroids alone for the treatment of high-risk acute graft versus host disease. | 2 | Allogenic | Acute Graft Versus Host Disease, | USA | 60 |
| NCT05647213 | Autologous Induced Pluripotent Stem Cells of Cardiac Lineage for Congenital Heart Disease | Recruiting | To test the safety of lab-grown heart cells made from stem cells in subjects with congenital heart disease. | 1 | Autologous | Univentricular Heart, Congenital Heart Disease, Heart Failure NYHA Class III | USA | 50 |
| NCT06049342 | A Trial to Evaluate the Safety, Tolerability, and Efficacy of NCR100 Injection in the Treatment of Subjects With knee osteoarthritis (KOA) | Not yet recruiting | To investigate the safety and efficacy of NCR100 injection in subjects with KOA. | 1 | Allogenic | KOA | China | 12 |
| NCT06145711 | A Clinical Trial of Parkinson's Disease Treatment by hiPSCs Derived Dopaminergic Neural Precursor Cells | Not yet recruiting | To explore the safety of stem cell investigational drugs. | N/A | Autologous | Parkinson's Disease | China | 3 |
| NCT06245018 | A Trial to Evaluate the Safety and Preliminary Efficacy of iNK in the Treatment of Subjects With Solid Tumor | Not yet recruiting | To investigate the safety, efficacy of iNK in subjects with solid tumor. | N/A | ? | Solid tumors | China | 50 |
| NCT06255028 | A Study of CNTY-101 in Participants With Refractory B Cell-mediated Autoimmune Diseases (CALiPSO-1) | Recruiting | To evaluate the safety and efficacy of CNTY-101 in participants with refractory B cell-mediated autoimmune diseases. | 1 | Allogeneic | Systemic Lupus Erythematosus, Lupus Nephritis | USA | 30 |
| NCT06299033 | A Safety and Tolerability Study of Human Forebrain Neural Progenitor Cells Injection (hNPC01) in Subjects With Chronic Ischemic Stroke | Recruiting | To evaluate the safety and tolerability of hNPC01 in chronic ischemic stroke. | 1 | Allogeneic | Chronic Ischemic Stroke | China | 21 |
| NCT06321198 | A Trial to Evaluate the Safety and Preliminary Efficacy of iMesenchymal Stromal Cells (iMSC) in Subjects With SR-aGVHD | Recruiting | To evaluate the safety and preliminary efficacy of iMSC in subjects with SR-aGVHD. | N/A | ? | Steroid-refractory Acute Graft-versus-host Disease (SR-aGVHD) | China | 12 |
| NCT06342986 | Intraperitoneal FT536 in Recurrent Ovarian, Fallopian Tube, and Primary Peritoneal Cancer | Recruiting | ? | 1 | Allogeneic | Gynecologic Cancer, Ovarian Cancer, Fallopian Tube Cancer | USA | 33 |
| NCT06344026 | Phase 1/2a Study of ANPD001 in Parkinson Disease | Enrolling | To test the safety and tolerability of injecting ANPD001 cells that will mature into dopamine-producing cells into the brain of participants with Parkinson Disease. | 1 | Autologous | Parkinson Disease | USA | 9 |
| NCT06394232 | Safety & Efficacy of Eyecyte-RPE™ in Patients With Geographic Atrophy (GA) Secondary to Dry Age-related Macular Degeneration (d-AMD) | Recruiting | To evaluate the safety and efficacy of novel stem cell formulation in patients having GA Secondary to d-AMD. | 1,2 | Allogeneic | Retinal Disease, Macular Degeneration, Age-Related Macular Degeneration | India | 54 |
| NCT06422208 | Autologous iPSC-Derived Dopamine Neuron Transplantation for Parkinson's Disease | Enrolling | To test the safety of injecting the investigational cell product into the brain of subjects with Parkinson's disease. | 1 | Autologous | Parkinson Disease | USA | 6 |
| NCT06482268 | Transplantation of Human iPS Cell-derived Dopaminergic Progenitors (CT1-DAP001) for Parkinson's Disease (Phase I/II) | Recruiting | To evaluate the safety and efficacy of transplantation of CT1-DAP001 into the corpus striatum in patients with Parkinson's disease. | 1 | Allogeneic | Parkinson Disease | USA | 7 |

**Other studies**

| **Trial ID** | **Title** | **Status** | **Description** | **Phase** | **Type of cell** | **Condition** | **Country** | **Enrolment** |
| --- | --- | --- | --- | --- | --- | --- | --- | --- |
| (Mandai et al., 2017) | Autologous Induced Stem-Cell–Derived Retinal Cells for Macular Degeneration | Completed | To assess the feasibility of transplanting a sheet of retinal pigment epithelial (RPE) cells differentiated from induced pluripotent stem cells (iPSCs) in a patient with neovascular age-related macular degeneration | Case report | Autologous | Neovascular age-related macular degeneration | Japan | 1 |
| UMIN000026003 | HLA-Matched Allogeneic iPS Cells-Derived RPE Transplantation for Macular Degeneration | Completed | To assess the safety and the immune reactions after iPS cells-derived retinal pigment epithelium (iPS-RPE) transplantation | – | Allogenic (HLA-homozygote) | Wet AMD with an atrophic RPE lesion | Japan | 5 |
| JPRN-jRCTa050210178 | Clinical Research of allogeneic iPSC-RPE cell strip transplantation for RPE impaired disease | Recruiting | – | Phases ½ | Allogenic | RPE impaired disease | Japan | 50 |
| UMIN000033564 | Kyoto Trial to Evaluate the Safety and Efficacy of iPSC-derived dopaminergic progenitors in the treatment of Parkinson's Disease | Completed | To evaluate the safety and efficacy of transplantation of human induced pluripotent stem cell-derived dopaminergic progenitors into the corpus striatum in patients with Parkinson's disease | Phases ½ | – | Parkinson's disease | Japan | 7 |
| (Schweitzer et al., 2020) | Personalized iPSC-Derived Dopamine Progenitor Cells for Parkinson’s Disease | Completed | – | Case report | Autologous | Idiopathic Parkinson’s disease | USA | 1 |
